# The impact of PM_2.5_ and its constituents on gestational diabetes mellitus: a retrospective cohort study

**DOI:** 10.1186/s12889-024-19767-1

**Published:** 2024-08-19

**Authors:** Weiqi Liu, Haidong Zou, Weiling Liu, Jiangxia Qin

**Affiliations:** 1 Department of Clinical Laboratory, The Maternal and Children Health Care Hospital (Huzhong Hospital) of Huadu, Guangzhou, 510800 Guangdong People’s Republic of China; 2 Department of Obstetrics, The Maternal and Children Health Care Hospital (Huzhong Hospital) of Huadu, Guangzhou, 510800 Guangdong People’s Republic of China; 3 Department of Clinical Laboratory, Foshan Fosun Chancheng Hospital, Foshan, 528000 Guangdong People’s Republic of China

**Keywords:** Black carbon, Fine particulate matter, Gestational diabetes mellitus, Odds ratios, Sulfate

## Abstract

**Background:**

There is increasing evidence that exposure to PM_2.5_ and its constituents is associated with an increased risk of gestational diabetes mellitus (GDM), but studies on the relationship between exposure to PM_2.5_ constituents and the risk of GDM are still limited.

**Methods:**

A total of 17,855 pregnant women in Guangzhou were recruited for this retrospective cohort study, and the time-varying average concentration method was used to estimate individual exposure to PM_2.5_ and its constituents during pregnancy. Logistic regression was used to assess the relationship between exposure to PM_2.5_ and its constituents and the risk of GDM, and the expected inflection point between exposure to PM_2.5_ and its constituents and the risk of GDM was estimated using logistic regression combined with restricted cubic spline curves. Stratified analyses and interaction tests were performed.

**Results:**

After adjustment for confounders, exposure to PM_2.5_ and its constituents (NO_3_^−^, NH_4_^+^, and OM) was positively associated with the risk of GDM during pregnancy, especially when exposure to NO_3_^−^ and NH_4_^+^ occurred in the first to second trimester, with each interquartile range increase the risk of GDM by 20.2% (95% CI: 1.118–1.293) and 18.2% (95% CI. 1.107–1.263), respectively. The lowest inflection points between PM_2.5_, SO_4_^2−^, NO_3_^−^, NH_4_^+^, OM, and BC concentrations and GDM risk throughout the gestation period were 18.96, 5.80, 3.22, 2.67, 4.77 and 0.97 µg/m^3^, respectively. In the first trimester, an age interaction effect between exposure to SO_4_^2−^, OM, and BC and the risk of GDM was observed.

**Conclusions:**

This study demonstrates a positive association between exposure to PM_2.5_ and its constituents and the risk of GDM. Specifically, exposure to NO_3_^−^, NH_4_^+^, and OM was particularly associated with an increased risk of GDM. The present study contributes to a better understanding of the effects of exposure to PM_2.5_ and its constituents on the risk of GDM.

**Supplementary Information:**

The online version contains supplementary material available at 10.1186/s12889-024-19767-1.

## Background

Gestational diabetes mellitus (GDM) is a common metabolic disorder of pregnancy, and its incidence has increased in recent years. It is estimated that GDM affects approximately 16.7% of pregnancies worldwide, affecting approximately 21 million live births, and in China, the prevalence of GDM has reached 8.6% [1]. GDM affects not only the health of pregnant women, [2–4] but also the potential occurrence of adverse pregnancy outcomes, including macrosomia and neonatal hypoglycaemia, and increases the long-term risk of diabetes in both mothers and children [5–7]. Therefore, to reduce the risk of GDM and its associated complications, it is particularly important to study the pathogenic factors of GDM.

The mechanisms through which fine particulate matter (PM_2.5_) exposure leads to GDM are not fully understood and may involve multiple pathways that increase the risk of GDM. Animal experiments by Xu J et al. have shown that PM_2.5_ exposure in mice induces oxidative stress mediated by nuclear factor erythroid 2 related factor 2 and activates inhibitory signaling pathways mediated by c-Jun N-terminal kinase, leading to hepatic insulin resistance (IR). [8] PM_2.5_ contains thousands of chemical constituents, with polycyclic aromatic hydrocarbons (PAHs) being the most prominent organic constituents. Research suggests that lipophilic PAHs may contribute to IR through methylation-mediated suppression of the insulin receptor substrate 2 gene. [9] Additionally, PM_2.5_ also interferes with the inflammatory response in visceral adipose tissue, lipid metabolism in hepatocytes and glucose metabolism in skeletal muscle by altering the CC-chemokine receptor 2 signalling pathway, further exacerbating insulin resistance. [10] An increasing number of studies suggest that exposure to PM_2.5_ is associated with an increased risk of developing diabetes [11–14]. According to a study of 395,927 pregnant women in southern California, exposure to ambient PM_2.5_ increases the likelihood of developing gestational diabetes mellitus (GDM) [15]. A case‒control study by Shen HN et al. [16] revealed that exposure to PM_2.5_ in early and mid-pregnancy increased the risk of GDM by 9% (95% CI 1.02‒1.17) and 7% (95% CI 1.01‒1.14), respectively. A positive association between PM_2.5_ exposure in the second trimester and GDM risk was found in a study of 2,078,669 people in Florida between 2005 and 2015 [17]. However, there is also evidence that exposure to PM_2.5_ is not associated with an increased risk of GDM. [18, 19] Therefore, the relationship between PM_2.5_ exposure and the risk of gestational diabetes is controversial and needs to be clarified by further large-scale studies.

PM_2.5_ is composed of a variety of substances, including sulfate (SO_4_^2−^), nitrate (NO_3_^−^), ammonium (NH_4_^+^), organic matter (OM), and black carbon (BC). The toxicity of PM_2.5_ constituents to people is variable. Wang X et al. [20] conducted a study on PM_2.5_ constituents and asthma in six low- and middle-income countries and found that ammonia may be the main cause of asthma. Li S et al. [21] conducted a large-scale epidemiological survey in Southwest China and showed that OM may be the main cause of the association between PM_2.5_ exposure and diabetes mellitus risk. BC and OM were found to be the PM_2.5_ constituents that are most strongly and consistently associated with cardiovascular mortality and morbidity. [22] However, evidence on the relationship between exposure to PM_2.5_ constituents and GDM risk is limited. Previous studies have focused on the relationship between PM_2.5_ exposure and GDM risk, and a further understanding of the relationship between exposure to different PM_2.5_ constituents and the risk of GDM could rationally explain which component is responsible for the relationship between PM_2.5_ exposure and GDM risk and provide new opportunities to reduce the burden of GDM associated with PM_2.5_ exposure.

To address the research needs in this area, this retrospective cohort study evaluated the association of exposure to PM_2.5_ and its constituents with the risk of GDM in a population from Guangzhou city, Guangdong Province, China, to provide a basis for the targeted prevention and control of PM_2.5_ constituents.

## Methods

### Study cohort

This retrospective study focused on pregnant women who visited the Maternal and Children Health Care Hospital of Huadu in Guangzhou between 2020 and 2022. This specialized hospital primarily serves pregnant women and children, and its services cover the entire Guangzhou territory. The data of the study participants were obtained from the electronic case management system of the hospital, and GDM diagnoses were made according to the ICD-10 classification criteria for participants with diagnosis code O24. Participants who met the following criteria were included in the study: lived in Guangzhou during pregnancy, had complete relevant data, were not pregnant with twins, had no history of diabetes or hypertension before pregnancy, and conceived naturally. Notably, as this study used deidentified information, it was not necessary to obtain informed consent. This study was approved by the Ethics Committee of t the Maternal and Child Health Hospital of Huadu District (No. 2024-001).

### Exposure to PM_2.5_ and its constituents

To obtain daily concentrations of PM_2.5_ and its constituents, including SO_4_^2−^, NO_3_^−^, NH_4_^+^, OM, and BC, at a spatial resolution of 10 km × 10 km, we used data from the Tracking Air Pollution in China (TAP) project. This dataset, accessible via the web portal (http://tapdata.org.cn), consolidates ground-level measurements from various publications and supplements them with satellite-derived estimates. The estimation process used aerosol optical depth (AOD) data in conjunction with the GEOS-Chem atmospheric chemistry transport model, as described by Liu et al. [23] The temperature and relative humidity data used in this study were obtained from a website (https://rp5.ru/), and the monitoring site used was Guangzhou Airport.

To assess the exposure concentrations of PM_2.5_ and its constituents for each study participant, we used the time-varying average concentration method. Specifically, since all participants lived in Guangzhou, we first collected daily average concentrations of PM_2.5_ and its constituents for the city. Using the average for the entire region and each participant’s gestational week and delivery date, we estimated their average exposure concentrations during the first trimester (1–13 gestational weeks, T1), the second trimester (14–28 gestational weeks, T2), and first to second trimester (T1 + T2).

### Covariates

Based on earlier studies [24, 25] and information obtained from electronic medical records, we selected potential confounders, including age, ethnicity, occupation type, marital status, blood type, nonprimiparous status, anaemia status, infant weight, preeclampsia status, vaginitis status, gestational hypertension status, thyroid disease status, temperature, and relative humidity. Participants self-reported their ethnicity (Han, Hui, Miao, Tujia, etc.), occupation type (employee, civil servant, professional, self-employed, farmer, unemployed, etc.), marital status (married, divorced), blood type (A, B, O, AB), and whether they were first-time mothers or had given birth to at least one child. Ethnicity was reclassified as Han or other; occupation type was reclassified as employed, self-employed, or other; and infant weight was classified as low birth weight (< 2500 g), normal birth weight (2500–4000 g), or macrosomia (> 4000 g) based on the recorded birth weight. Assessing exposure to temperature and relative humidity using the same methodology as for PM_2.5_ and its constituents.

### Diagnosis of GDM

According to the diagnostic criteria for GDM, [26, 27] all pregnant women underwent oral glucose tolerance tests after fasting for at least 8 h between the 24th and 28th weeks of pregnancy. During the test, the pregnant woman had to drink 300 ml of a solution containing 75 g of glucose within 5 min. Blood glucose levels were measured before, 1 h after, and 2 h after glucose ingestion. According to medical guidelines, the blood glucose levels of pregnant women should be kept below 5.1 mmol/L, 10.0 mmol/L and 8.5 mmol/L at these three times. If a pregnant woman’s blood glucose level meets or exceeds any of the above criteria, she will be diagnosed with GDM by a health care professional.

### Statistical analyses

We used chi-squared or nonparametric tests for baseline characteristics. and Spearman’s rank correlation test was used to assess the correlations between exposure to PM_2.5_ and its constituents. Logistic regression analyses were used to estimate the odds ratios (ORs) and 95% confidence intervals (95% CIs) associated with the development of GDM, adjusting for potential confounders, including age, ethnicity, occupation type, marital status, blood type, nonprimary status, anaemia status, infant weight, preeclampsia status, vaginitis status, gestational hypertension status, thyroid disease status, temperature, and relative humidity. We used a logistic regression combined with restricted cubic spline curves to assess the relationship between exposure to PM_2.5_ and its constituents and the risk of GDM, with the reference value (OR = 1) set at the 10th percentile and the nodes set at the 5th, 35th, 65th, and 95th percentiles of the concentrations of PM_2.5_ and its constituents. Furthermore, we conducted stratified analyses to evaluate the impact of exposure to PM_2.5_ and its constituents on GDM risk.

Statistical analyses were performed with STATA 16.0 (StataCorp, USA) and R 4.3.2 (Lucent Technologies, USA) using the “rcssci” and “autoReg” packages. A two-tailed *p* < 0.05 was considered to indicate statistical significance.

## Results

### Baseline characteristics

In total, 17,855 pregnant women were included in our study, and 22.14% of the participants had GDM. The median (P25, P75) age of the participants was 29 years (26 years, 33 years), and 14.86% of the pregnant women were of an advanced maternal age. The median exposure concentrations for PM_2.5_, SO_4_^2−^, and OM in the GDM group were greater than those in the non-GDM group, and the temperature and relative humidity in the GDM group were greater than those in the non-GDM group. Further details are shown in Table 1.

**Table 1:** The baseline characteristics of the participants, PM_2.5_ and its constituents, and meteorological factors (2020–2022)

Variable	Overall (*N* = 17855)	GDM		*p*-value
		Yes(*N* = 3236)	No (*N* = 14619)	
Age, *n* (%)				< 0.001
< 35 years	15,199 (85.12)	2444 (75.53)	12,755 (87.25)	
≥ 35 years	2656 (14.88)	792 (24.47)	1864 (12.75)	
Ethnicity^a^, *n* (%)				0.707
Han	17,467 (97.83)	3169 (97.93)	14,298 (97.80)	
other	388 (2.17)	67 (2.07)	321 (2.20)	
Occupation type^b^, *n* (%)				0.033
Employee	10,285 (57.60)	1798 (55.56)	8487 (58.05)	
Freelancer	1263 (7.07)	244 (7.54)	1019 (6.97)	
Other	6307 (35.32)	1194 (36.90)	5113 (34.98)	
Marital status, *n* (%)				0.015
Married	17,284 (96.80)	3155 (97.50)	14,129 (96.65)	
Unmarried	571 (3.20)	81 (2.50)	490 (3.35)	
Blood type, *n* (%)				0.131
Type A	4899 (27.44)	840 (25.96)	4059 (27.77)	
Type B	4471 (25.04)	850 (26.27)	3621 (24.77)	
Type O	7297 (40.87)	1327 (41.01)	5970 (40.84)	
Type AB	1188 (6.65)	219 (6.77)	969 (6.63)	
Nonprimary status, *n* (%)				< 0.001
No	10,063 (56.36)	1681 (51.95)	8382 (57.34)	
Yes	7792 (43.64)	1555 (48.05)	6237 (42.66)	
Anemia status, *n* (%)				< 0.001
No	9167 (51.34)	1866 (57.66)	7301 (49.94)	
Yes	8688 (48.66)	1370 (42.34)	7318 (50.06)	
Infant gender, *n* (%)				0.961
Male	9511 (53.27)	1722 (53.21)	7789 (53.28)	
Female	8344 (46.73)	1514 (46.79)	6830 (46.72)	
Infant weight, *n* (%)				< 0.001
≤ 2499 g	929 (5.20)	219 (6.77)	710 (4.86)	
2500–4000 g	16,574 (92.83)	2945 (91.01)	13,629 (93.23)	
>4000 g	352 (1.97)	72 (2.22)	280 (1.92)	
Preeclampsia status, *n* (%)				< 0.001
No	17,301 (96.90)	3074 (94.99)	14,227 (97.32)	
Yes	554 (3.10)	162 (5.01)	392 (2.68)	
Vaginitis status, *n* (%)				0.871
No	15,547 (87.07)	2821 (87.18)	12,726 (87.05)	
Yes	2308 (12.93)	415 (12.82)	1893 (12.95)	
Gestational hypertension status, *n* (%)				< 0.001
No	17,441 (97.68)	3116 (96.29)	14,325 (97.99)	
Yes	414 (2.32)	120 (3.71)	294 (2.01)	
Thyroid disease status, *n* (%)				0.597
No	16,824 (94.23)	3056 (94.44)	13,768 (94.18)	
Yes	1031 (5.77)	180 (5.56)	851 (5.82)	
Pollution, median (IQR)^c^				
PM_2.5_, (µg/m^3^)	24.63 (19.24, 30.38)	24.64 (19.24, 30.38)	24.55 (19.2, 30.21)	0.854
SO_4_^2−^, (µg/m^3^)	4.78 (3.88, 5.83)	4.78 (3.88, 5.82)	4.75 (3.88, 5.86)	0.521
NO_3_^−^, (µg/m^3^)	3.64 (2.29, 4.58)	3.65 (2.30, 4.58)	3.59 (2.28, 4.57)	0.454
NH_4_^+^, (µg/m^3^)	2.77 (1.84, 3.40)	2.77 (1.84, 3.41)	2.76 (1.83, 3.38)	0.587
OM, (µg/m^3^)	6.34 (5.14, 8.02)	6.34 (5.14, 8.02)	6.34 (5.14, 8.02)	0.667
BC, (µg/m^3^)	1.27 (1.10, 1.63)	1.27 (1.10, 1.63)	1.28 (1.10, 1.63)	0.399
Meteorological factors, median (IQR)^c^				
Temp, (℃)	22.49 (19.69, 25.73)	22.46 (19.65, 25.71)	22.64 (19.9, 25.83)	0.032
RH, (%)	77.16 (75.29, 80.19)	77.12 (75.26, 80.16)	77.55 (75.4, 80.39)	< 0.001

^a^ Han, Hui, Miao, Tujia, etc

^b^ employee, civil servant, professional, self-employed, farmer, unemployed, etc

^c^ median (IQR) for exposure during the first to second trimester (T1 + T2)

*IQR*, interquartile range. *PM*_*2.5*_, fine particulate matter. *SO*_*4*_^*2−*^, sulfate. *NO*_*3*_^*−*^, nitrate. *NH*_*4*_^*+*^, ammonium. *OM*, organic matter. *BC*, black carbon. *Temp*, temperature. *RH*, relative humidity

### Correlation analysis of PM_2.5_, SO_4_^2−^, NO_3_^−^, NH_4_^+^, OM, and BC concentrations

Table 2 shows the concentrations of PM_2.5_, SO_4_^2−^, NO_3_^−^, NH_4_^+^, OM, and BC during the study period. There was a strong correlation among PM_2.5_, SO_4_^2−^, NO_3_^−^, NH_4_^+^, OM, and BC concentrations (Spearman’s correlation coefficient > 0.8). To ensure that the results of the correlation analysis were not affected by outliers, we performed a sensitivity analysis. Specifically, we chose the 95th percentile of PM_2.5_ concentration as a threshold to exclude extreme values from the dataset and recalculated the correlation coefficients. We found that the correlation coefficients between PM_2.5_ and its components did not significantly change after removing the extreme values (Table S1).

**Table 2:** Spearman correlation coefficients between the mean daily concentrations of PM_2.5_, SO_4_^2−^, NO_3_^−^, NH_4_^+^, OM, and BC during the study period (2020 and 2022)

	PM_2.5_	SO_4_^2−^	NO_3_^−^	NH_4_^+^	OM	BC
PM_2.5_	1.000					
SO_4_^2−^	0.987	1.000				
NO_3_^−^	0.944	0.914	1.000			
NH_4_^+^	0.965	0.941	0.996	1.000		
OM	0.995	0.991	0.927	0.951	1.000	
BC	0.983	0.992	0.895	0.925	0.994	1.000

*PM*_*2.5*_, fine particulate matter. *SO*_*4*_^*2−*^, sulfate. *NO*_*3*_^*−*^, nitrate. *NH*_*4*_^*+*^, ammonium. *OM*, organic matter. *BC*, black carbon

All correlations are significant at *P* < 0.001

### Relationship between PM_2.5_, SO_4_^2−^, NO_3_^−^, NH_4_^+^, OM, and BC exposure and GDM risk

Table 3 shows the associations between exposure to PM_2.5_, SO_4_^2−^, NO_3_^−^, NH_4_^+^, OM, and BC and the risk of GDM. After adjusting for confounding factors, in the first trimester, the ORs per Interquartile range (IQR) increase in PM_2.5_, SO_4_^2−^, NO_3_^−^, NH_4_^+^, OM, and BC concentrations were associated with an increase in the risk of GDM by 9.2% (95% CI: 1.034–1.154), 8. 6% (95% CI: 1.035–1.140), 11.6% (95% CI: 1.034–1.023), 11.1% (95% CI: 1.037–1.190), 9.7% (95% CI: 1.040–1.158), and 8.5% (95% CI: 1.039–1.134), respectively. Exposure to PM_2.5_, NO_3_^−^, NH_4_^+^, and OM in the second trimester and exposure to PM_2.5_, SO_4_^2−^, NO_3_^−^, NH_4_^+^, OM, and BC from the first to second trimester also increased the risk of GDM.

**Table 3:** ORs and 95% CIs of the associations between GDM risk and each IQR increase in PM_2.5_, SO_4_^2−^, NO_3_^−^, NH_4_^+^, OM and BC concentrations

Variable	Crude		Adjusted^a^	
	OR (95%CI)	*p*-value	OR (95%CI)	*p*-value
**T1**				
PM_2.5_	0.994 (0.961–1.029)	0.736	1.092 (1.034–1.154)	0.002
SO_4_^2−^	1.013 (0.980–1.049)	0.429	1.086 (1.035–1.140)	0.001
NO_3_^−^	0.985 (0.952–1.019)	0.381	1.116 (1.034–1.023)	0.005
NH_4_^+^	0.987 (0.954–1.022)	0.465	1.111 (1.037–1.190)	0.003
OM	0.999 (0.966–1.034)	0.970	1.097 (1.040–1.158)	0.001
BC	1.027 (0.992–1.062)	0.132	1.085 (1.039–1.134)	< 0.001
**T2**				
PM_2.5_	0.999 (0.966–1.034)	0.976	1.082 (1.014–1.154)	0.017
SO_4_^2−^	1.000 (0.967–1.034)	1.000	1.050 (0.994–1.108)	0.079
NO_3_^−^	0.995 (0.961–1.029)	0.758	1.141 (1.047–1.242)	0.002
NH_4_^+^	0.996 (0.962–1.030)	0.810	1.120 (1.036–1.211)	0.004
OM	1.005 (0.972–1.040)	0.761	1.086 (1.023–1.153)	0.007
BC	0.999 (0.965–1.033)	0.938	1.042 (0.988–1.097)	0.130
**T1 + T2**				
PM_2.5_	0.987 (0.954–1.021)	0.454	1.105 (1.046–1.167)	< 0.001
SO_4_^2−^	1.002 (0.968–1.037)	0.910	1.091 (1.039–1.144)	< 0.001
NO_3_^−^	0.986 (0.953–1.020)	0.424	1.202 (1.118–1.293)	< 0.001
NH_4_^+^	0.991 (0.958–1.026)	0.611	1.182 (1.107–1.263)	< 0.001
OM	0.995 (0.961–1.029)	0.761	1.103 (1.046–1.162)	< 0.001
BC	1.009 (0.976–1.044)	0.592	1.096 (1.046–1.149)	< 0.001

^a^ Adjusted for age, ethnicity, occupation type, marital status, blood type, nonprimary status, anaemia status, infant weight, preeclampsia status, vaginitis status, gestational hypertension status, thyroid disease status, temperature, and relative humidity

*T1*, the first trimester. *T2*, the second trimester. *T1 + T2*, the first to second trimester

*PM*_*2.5*_, fine particulate matter. *SO*_*4*_^*2−*^, sulfate. *NO*_*3*_^*−*^, nitrate. *NH*_4_^*+*^, ammonium. *OM*, organic matter. *BC*, black carbon

**Fig. 1:**
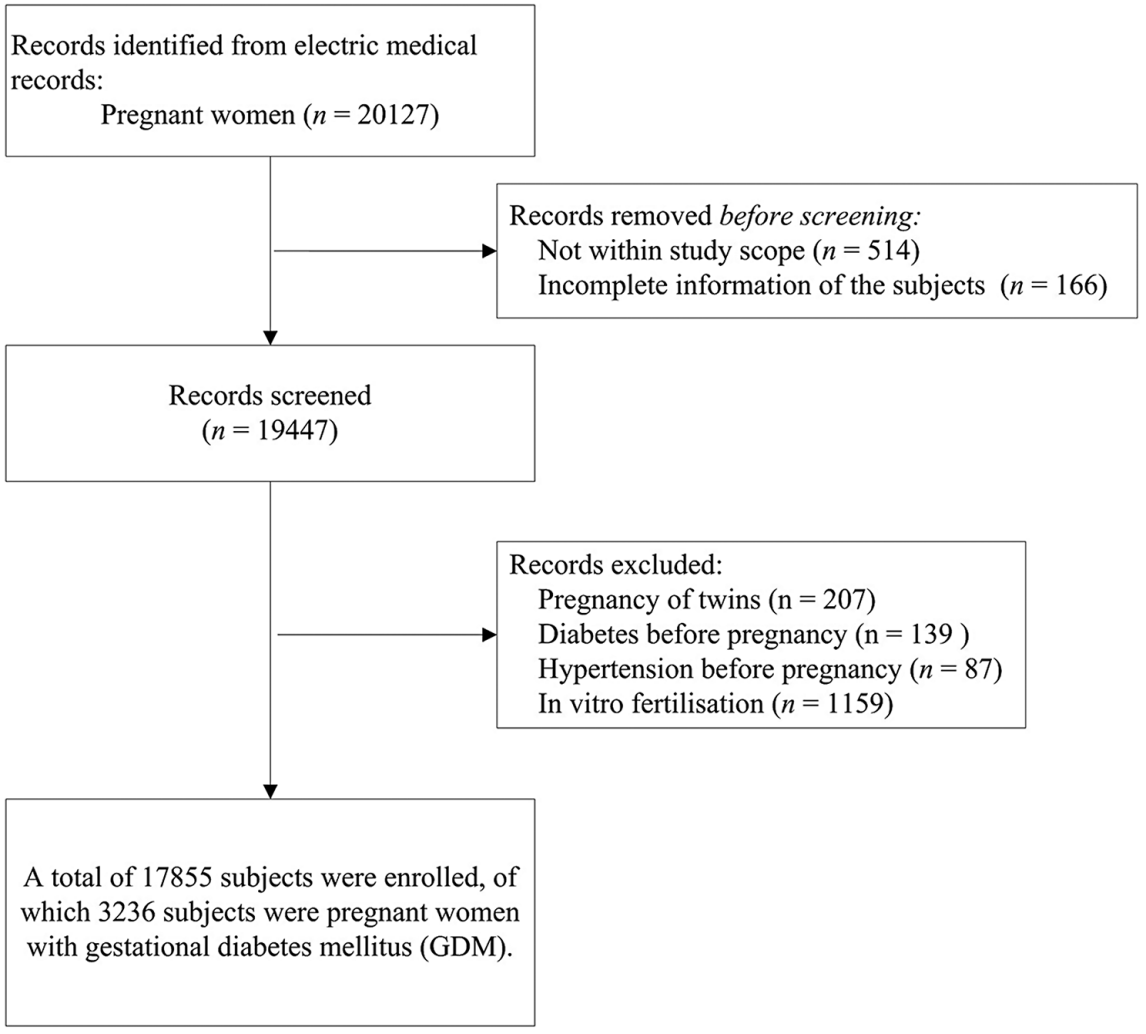
Flowchart of participant screening

**Fig. 2:**
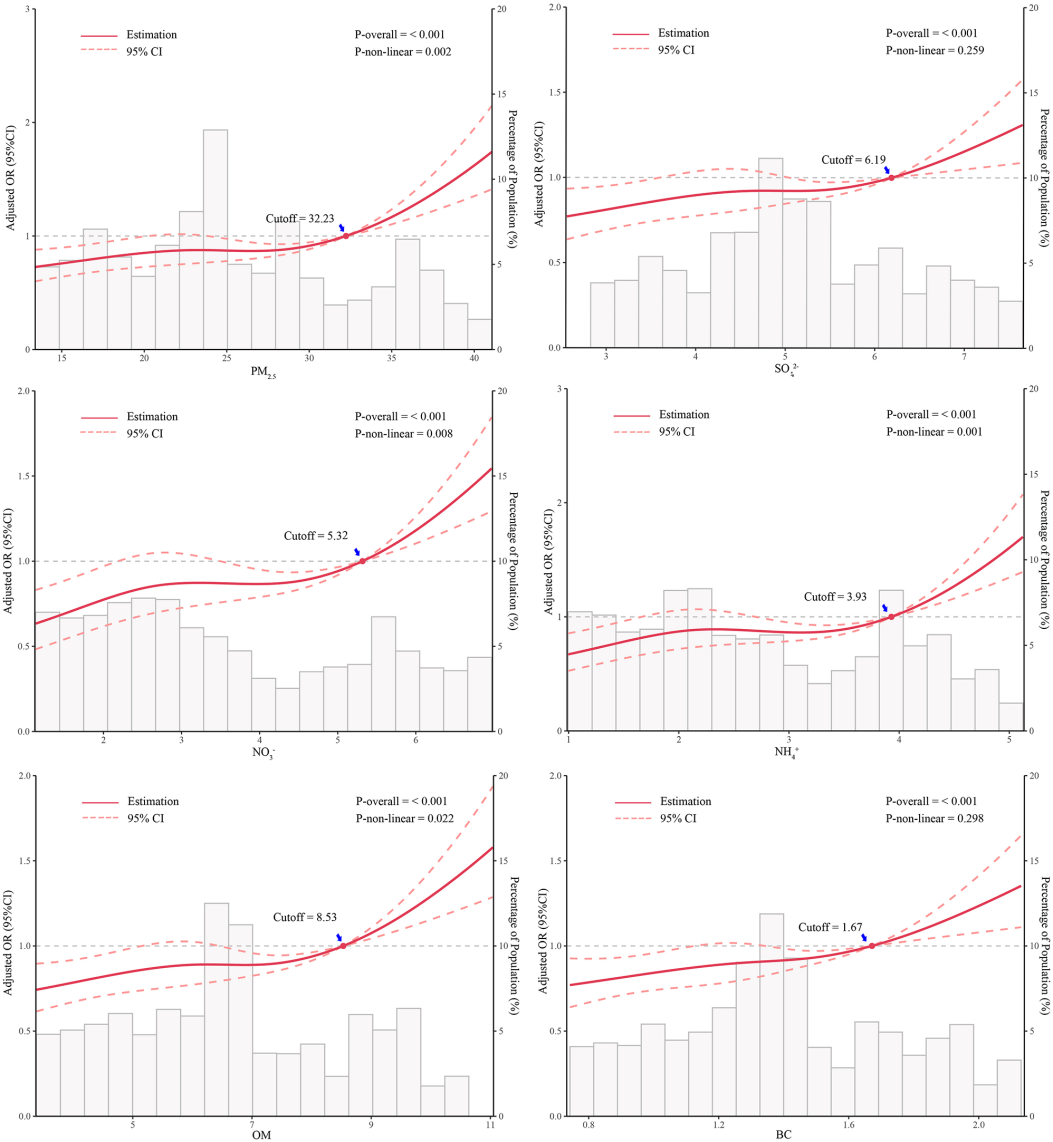
Association between predicted exposure to PM_2.5_ and its constituents during the first trimester and GDM risk. The solid line indicates the OR, and the dashed area indicates the 95% CI. The reference point is the lowest value for PM_2.5_ and its constituents, and the nodes are at the 5th, 35th, 65th, and 95th percentiles for PM_2.5_ and its constituents

After adjusting for confounders, we found that the inflection points between PM_2.5_, OM, and BC concentrations and GDM risk were lowest in the second trimester, at 18.96, 4.77 and 0.97 µg/m^3^, respectively. The inflection points between SO_4_^2−^, NO_3_^−^ and NH_*4*_^+^concentrations and GDM risk were lowest in the first to second trimester, at 5.80, 3.22 and 2.67 µg/m^3^, respectively. In addition, a nonlinear relationship between PM_2.5_, NO_3_^−^, NH_4_^+^, and OM exposure and GDM risk was observed only in the first trimester (p values for nonlinearity of 0.002, 0.008, 0.001 and 0.022, respectively) (Figs. 1, 2 Figure S1-S2).

### Subgroup analysis

**Fig. 3:**
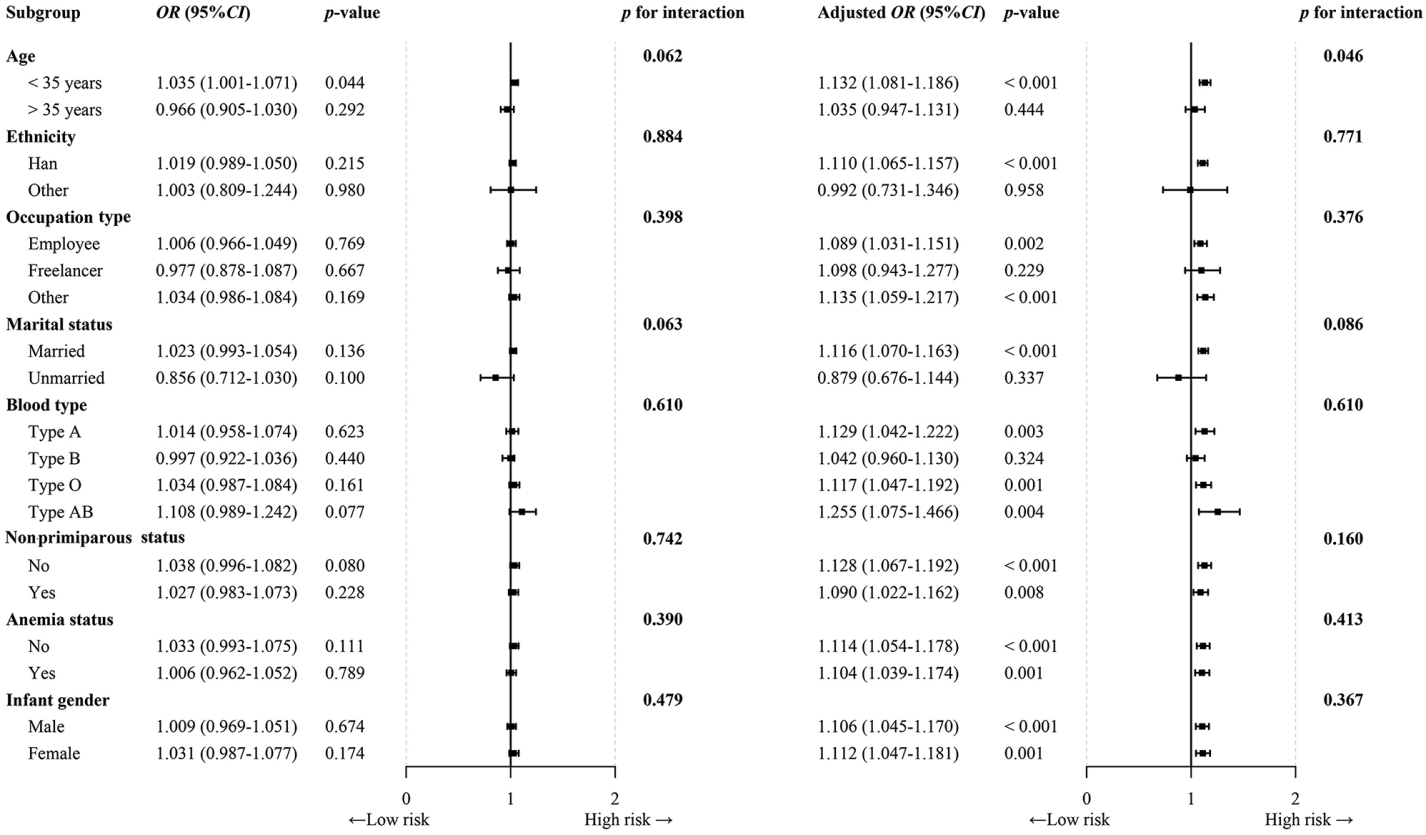
Forest plot of subgroup analysis of the relationship between SO_4_^2−^ exposure in the first trimester and GDM risk

To evaluate the association between exposure to PM_2.5_ and its constituents and GDM risk, stratified and interaction analyses of the study participants’ age, ethnicity, occupation type, marital status, blood type, nonprimiparous status, anaemia status and infant sex were performed. In the first trimester, significant associations between PM_2.5_, SO_4_^2−^, NO_3_^−^, NH_4_^+^, OM and BC exposure and GDM risk were observed in the nonprimiparous, anaemic and infant sex subgroups (*p* < 0.05) (Table 4; Fig. 3, Table S2-S5). A similar pattern of increased GDM risk was found in the second trimester and the first to second trimester subgroups. Details of the exposure effect sizes for the second trimester subgroup are given in Tables S6-S11. The exposure effect sizes for the first to second trimester subgroup are presented in Tables S12-S17. In addition, an interaction by age subgroup was observed only between exposure to SO_4_^2−^, OM and BC in the first trimester and GDM risk (p values for the interaction were 0.046, 0.046 and 0.044, respectively).

**Table 4:** Subgroup analysis of the association between PM_2.5_ exposure in the first trimester and GDM risk

Subgroup	Crude				Ajuested^a^		
	OR(95%CI)	*p*-value	*p* for interaction		OR(95%CI)	*p*-value	*p* for interaction
Age			0.083				0.052
< 35 years	1.004 (0.998–1.010)	0.245			1.032 (1.022–1.043)	< 0.001	
≥ 35 years	0.992 (0.981–1.004)	0.177			1.011 (0.992–1.031)	0.252	
Ethnicity			0.694				0.688
Han	1.001 (0.996–1.006)	0.677			1.027 (1.018–1.037)	< 0.001	
Other	0.993 (0.956–1.032)	0.735			1.001 (0.939–1.068)	0.968	
Occupation type			0.455				0.592
Employee	0.999 (0.992–1.007)	0.830			1.025 (1.012–1.037)	< 0.001	
Freelancer	0.992 (0.973–1.011)	0.387			1.021 (0.988–1.055)	0.221	
Other	1.003 (0.995–1.012)	0.421			1.031 (1.015–1.046)	< 0.001	
Marital status			0.115				0.131
Married	1.002 (0.996–1.007)	0.553			1.028 (1.019–1.038)	< 0.001	
Unmarried	0.975 (0.943–1.008)	0.133			0.976 (0.922–1.033)	0.408	
Blood type			0.685				0.633
Type A	1.001 (0.991–1.011)	0.891			1.032 (1.015–1.050)	< 0.001	
Type B	0.994 (0.984–1.004)	0.237			1.013 (0.995–1.031)	0.163	
Type O	1.003 (0.995–1.012)	0.416			1.028 (1.013–1.042)	< 0.001	
Type AB	1.014 (0.994–1.035)	0.176			1.056 (1.020–1.092)	0.002	
Nonprimary status			0.494				0.122
No	1.005 (0.997–1.012)	0.215			1.031 (1.019–1.043)	< 0.001	
Yes	1.001 (0.993–1.009)	0.813			1.023 (1.009–1.038)	0.002	
Anemia status			0.373				0.386
No	1.003 (0.996–1.011)	0.341			1.028 (1.015–1.041)	< 0.001	
Yes	0.999 (0.991–1.007)	0.728			1.026 (1.013–1.040)	< 0.001	
Infant gender			0.260				0.199
Male	0.998 (0.991–1.005)	0.620			1.024 (1.012–1.037)	< 0.001	
Female	1.004 (0.997–1.012)	0.281			1.030 (1.016–1.043)	< 0.001	

^a^ Adjusted for age, ethnicity, occupation type, marital status, blood type, nonprimary status, anaemia status, infant weight, preeclampsia status, vaginitis status, gestational hypertension status, thyroid disease status, temperature, and relative humidity

## Discussion

In this study, we found that exposure to the air pollutant PM_2.5_ and its constituents (SO_4_^2−^, NO_3_^−^, NH_4_^+^, OM and BC) is positively associated with an increased risk of GDM. In addition, nonlinear associations were found between PM_2.5_, NO_3_^−^, NH_4_^+^, OM exposure during the first trimester and GDM risk, while subgroup analyses revealed age interactions between exposure to SO_4_^2−^, OM and BC during the first trimester and GDM risk.

Numerous epidemiological studies have consistently revealed a correlation between exposure to PM_2.5_ and the risk of GDM, [28–30] which is consistent with the findings of this study. Tang et al. [31] analysed 13 studies (including 9 retrospective studies, 3 prospective studies and 1 case‒control study) and found that PM_2.5_ exposure in the second trimester was associated with an increased risk of GDM (OR 1.07, 95% CI 1.00 to 1.13), while PM_2.5_ exposure in the first trimester did not increase the risk of GDM (OR 1.01; 95% CI 0.96 to 1.07). A retrospective cohort study conducted in Shanghai, China, from 2014 to 2016 revealed that a 10 µg/m^3^ increase in PM_2.5_ exposure during the first trimester, second trimester, and first to second trimester increased the risk of GDM by 9% (95% CI: 1.02, 1.16), 9% (95% CI: 1.03, 1.16), and 15% (95% CI: 1.04, 1.28), respectively. [32] However, a study from Hebei, China, showed that PM_2.5_ exposure in the first trimester, second trimester, or first to second trimester did not increase the risk of GDM. [33] The results of this study showed that exposure to PM_2.5_ increased the risk of GDM by 9.2% (95% CI: 1.034–1.154), 8.2% (95% CI: 1.014–1.154), and 10.5% (95% CI: 1.046–1.167) in the first, second, and first to second trimester, respectively. This finding is consistent with a previous study conducted in Foshan city, Guangdong Province, from 2015 to 2019, which was a birth cohort study. The results showed that exposure to PM_2.5_ during the first, second, and first to second trimester increased the risk of GDM [34]. This may be due to the proximity of Foshan to Guangzhou and their similar geographical and climatic conditions. Such similarities could result in comparable sources, concentrations and compositions of PM_2.5_ pollution in both areas, leading to consistent research results between the two locations. In addition, similarities in residents’ lifestyles, dietary habits and other factors may contribute to similar sensitivities to PM_2.5_ exposure and susceptibility to GDM, further explaining the consistency of the research findings.

Strong seasonal and regional variations in PM_2.5_ constituents were suggested by Bell et al. [35] However, it is still unclear which PM_2.5_ constituents have the greatest effect on GDM risk, and research on the association between exposure to PM_2.5_ constituents and the risk of GDM remains limited. A cross-sectional survey conducted in 55 hospitals across 24 provinces in China from 2015 to 2016, with a total of 54,517 participants, revealed that organic compounds, black carbon, and nitrate may be the main causes behind the occurrence of GDM. [36] A retrospective cohort study conducted in the United States between 2002 and 2008 involving 201,015 participants revealed that each IQR increase in nitrate exposure during the first trimester was associated with a 5% (95% CI: 1.02–1.09) increased risk of GDM. However, exposure to elemental carbon, organic compounds, ammonium ions and sulfate did not increase the risk of GDM. [37] A recent meta-analysis of 31 eligible cohort studies revealed that second-trimester BC exposure and first-trimester NO_3_^−^ exposure increased the risk of GDM, with RRs of 1.128 (1.032–1.231) and 1.128 (1.032–1.231), respectively. A recent meta-analysis of 31 eligible cohort studies revealed that NO_3_^−^ exposure in the first trimester and BC exposure in the first to second trimester increased the risk of GDM by 5.6% (95% CI: 1.008–1.107) and 18.5% (95% CI: 1.026–1.368), respectively [38]. This finding is not entirely consistent with our findings in this retrospective cohort study, which revealed that although SO_4_^2−^ and BC exposure in the second trimester was negatively associated with GDM risk, SO_4_^2−^, NO_3_^−^, NH_4_^+^, OM, and BC exposure in other exposure windows were positively associated with GDM risk. The reason for this inconsistency may be due to significant variations in the levels of exposure to PM_2.5_ and its constituents in different countries and regions, as well as significant differences in the methods used to assess the exposure levels of the study participants.

Previous studies on the relationship between exposure to PM_2.5_ and its constituents and the risk of GDM have focused on risk assessment and exposure windows, [25, 39, 40]while the critical concentrations defining the association between these variables have been less explored. This study provides clearer evidence for the prevention of GDM in individuals with exposure to PM_2.5_ and its constituents by analysing the cut-off values of PM_2.5_ and its constituents associated with the occurrence of GDM. This study also provides a more precise basis for targeted interventions and policy development. In addition, we investigated the potential impacts of age, ethnicity, occupation type, marital status, blood type, nonprimiparous status, anaemia status, and infant sex. Our findings revealed a statistically significant age interaction between exposure to SO_4_^2−^, OM, and BC during the first trimester and the risk of GDM. Our results revealed a statistically significant age interaction effect between SO_4_^2−^, OM and BC exposure in the first trimester and GDM risk. This may be due to several factors. First, pregnant women of different ages have marked physiological differences, such as variations in metabolic rate, hormone levels and organ function, which may lead to different sensitivities to PM_2.5_ constituents. Second, with increasing age, prolonged exposure to environmental pollutants and the adoption of unhealthy lifestyles may increase the susceptibility of pregnant women to air pollutants in early pregnancy, thereby increasing the risk of GDM. Third, differences in prenatal nutrition, health care, work and family stress among age groups may differentially affect pregnant women’s susceptibility to air pollution. Finally, age-related changes in the immune system may lead to different immune responses to air pollutants in pregnant women. Such differences could increase the susceptibility of certain age groups to the effects of air pollutants, thereby increasing the likelihood of GDM.

There are a number of advantages to this study. First, the study population consists of pregnant women from Guangzhou, a large city in China, with a large sample size covering all 11 administrative districts of the city, which enhances the generalisability and applicability of the results. Second, we used logistic regression combined with restricted cubic splines, a method that allows us to accurately capture the exposure-response relationship and its non-linear effects. Finally, we adjusted the analysis for various confounding factors, such as age, ethnicity, occupation, marital status and blood group, and conducted subgroup analyses to explore heterogeneity among different subgroups. These measures increase the credibility of the results and provide new directions for future research. However, several limitations of this study need to be considered. First, there is a potential risk of exposure misclassification, as individual mobility was not taken into account during the exposure assessment, which may have affected the accuracy of the exposure estimates. Second, the cut-off for defining the onset of GDM in our study population was set at 28 weeks gestation rather than the clinically meaningful threshold of 24 weeks. This extended timeframe may have introduced ambiguity, potentially weakening the directness and clarity of the association between exposures and outcomes. Additionally, this study used a spatial resolution of 10 km x 10 km to estimate exposure to PM2.5 and its components, and the low spatial resolution of the exposure assessment may not be fine enough in some areas, especially localised urban pollution hotspots, which may affect the precision of the exposure estimates for the study population.

## Conclusion

Our results suggest that exposure to SO_4_^2−^ and BC during mid-pregnancy is negatively associated with GDM risk, whereas exposure to PM_2.5_ and its constituents during other windows is positively associated with an increased risk of GDM, adding to the evidence on the effects of exposure to PM_2.5_ and its constituents on the development of GDM. Furthermore, we identified thresholds for the effects of exposure to PM_2.5_ and its constituents on the risk of GDM during different exposure periods. These results have important implications for the prevention of GDM and call for further research to confirm our findings and elucidate the underlying mechanisms involved.

### Electronic supplementary material

Below is the link to the electronic supplementary material.

Supplementary Material 1

## Data Availability

No datasets were generated or analysed during the current study.
