# Supplementary material for: The impact of PM2.5 and its constituents on gestational diabetes mellitus: a retrospective cohort study
Source: BMC Public Health. 2024 Aug 19;24:2249. doi: 10.1186/s12889-024-19767-1 (PMC11334325; doi:10.1186/s12889-024-19767-1)
Supplement: Supplementary file 1 — Supplementary Material 1 [file 12889_2024_19767_MOESM1_ESM.docx]

Supplementary table

[Figure S1 1](#_Toc173328991)

[Figure S2 2](#_Toc173328992)

[Table S1 Adjusted Correlation Coefficients After PM_2.5_ Outlier Removal 3](#_Toc173328993)

[Table S2 Subgroup analysis of the association between NO_3_^-^ exposure in the first trimester and GDM risk 4](#_Toc173328994)

[Table S3 Subgroup analysis of the association between NH_4_^+-^ exposure in the first trimester and GDM risk 5](#_Toc173328995)

[Table S4 Subgroup analysis of the association between OM exposure in the first trimester and GDM risk 6](#_Toc173328996)

[Table S5 Subgroup analysis of the association between BC exposure in the first trimester and GDM risk 7](#_Toc173328997)

[Table S6 Subgroup analysis of the association between PM_2.5_ exposure in the second trimester and GDM risk 8](#_Toc173328998)

[Table S7 Subgroup analysis of the association between SO_4_^2-^ exposure in the second trimester and GDM risk 9](#_Toc173328999)

[Table S8 Subgroup analysis of the association between NO_3_^-^ exposure in the second trimester and GDM risk 10](#_Toc173329000)

[Table S9 Subgroup analysis of the association between NH_4_^+^ exposure in the second trimester and GDM risk 11](#_Toc173329001)

[Table S10 Subgroup analysis of the association between OM exposure in the second trimester and GDM risk 12](#_Toc173329002)

[Table S11 Subgroup analysis of the association between BC exposure in the second trimester and GDM risk 13](#_Toc173329003)

[Table S12 Subgroup analysis of the association between PM_2.5_ exposure in the first to second trimester and GDM risk 14](#_Toc173329004)

[Table S13 Subgroup analysis of the association between SO_4_^2-^ exposure in the first to second trimester and GDM risk 15](#_Toc173329005)

[Table S14 Subgroup analysis of the association between NO_3_^-^ exposure in the first to second trimester and GDM risk 16](#_Toc173329006)

[Table S15 Subgroup analysis of the association between NH_4_^+^ exposure in the first to second trimester and GDM risk 17](#_Toc173329007)

[Table S16 Subgroup analysis of the association between OM exposure in the first to second trimester and GDM risk 18](#_Toc173329008)

[Table S17 Subgroup analysis of the association between BC exposure in the first to second trimester and GDM risk 19](#_Toc173329009)


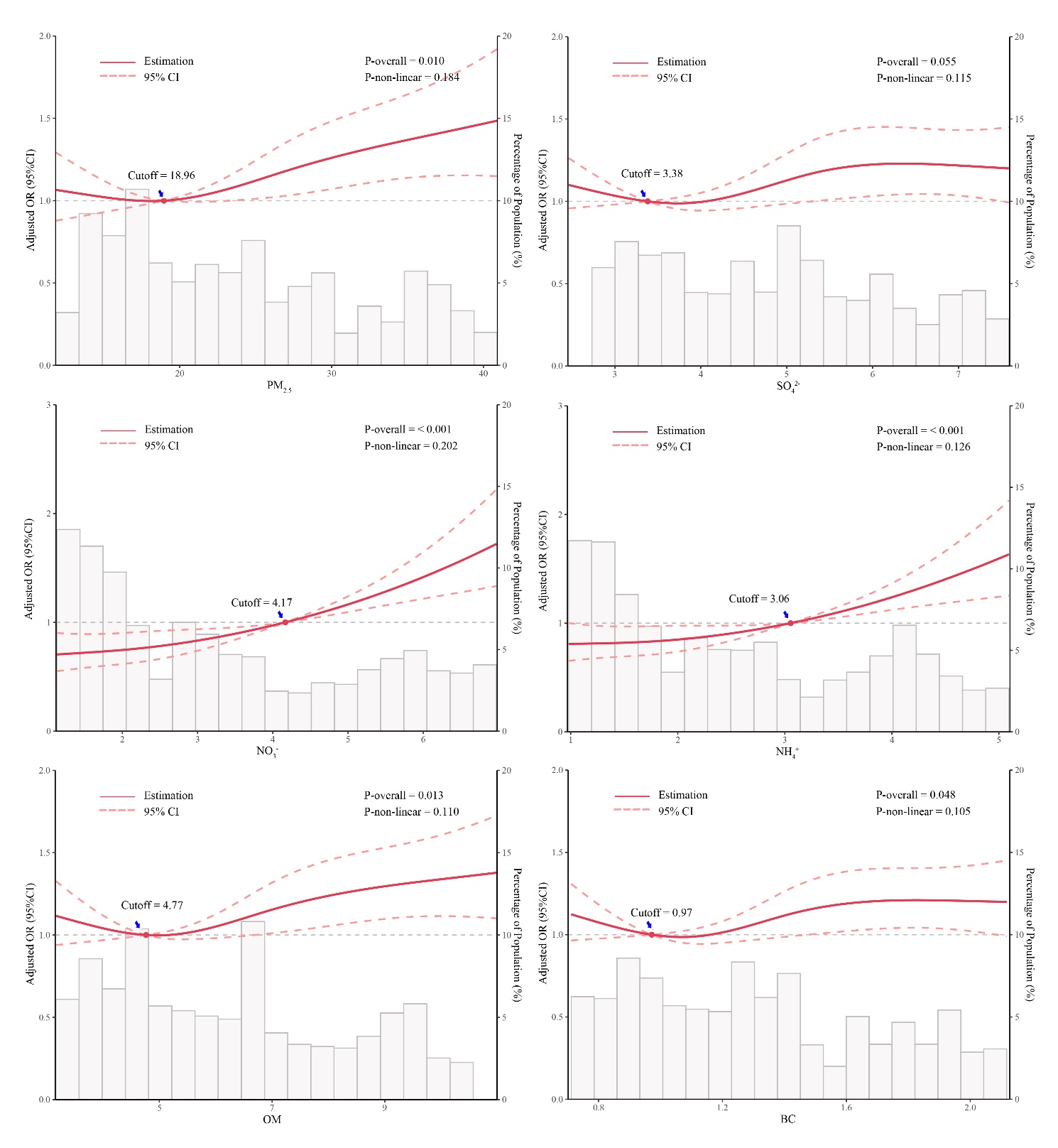


Figure S1 Association between predicted exposure to PM_2.5_ and its constituents during the second trimester and GDM risk. The solid line indicates the OR, and the dashed area indicates the 95% CI. The reference point is the lowest value for PM_2.5_ and its constituents, and the nodes are at the 5th, 35th, 65th, and 95th percentiles for PM_2.5_ and its constituents.


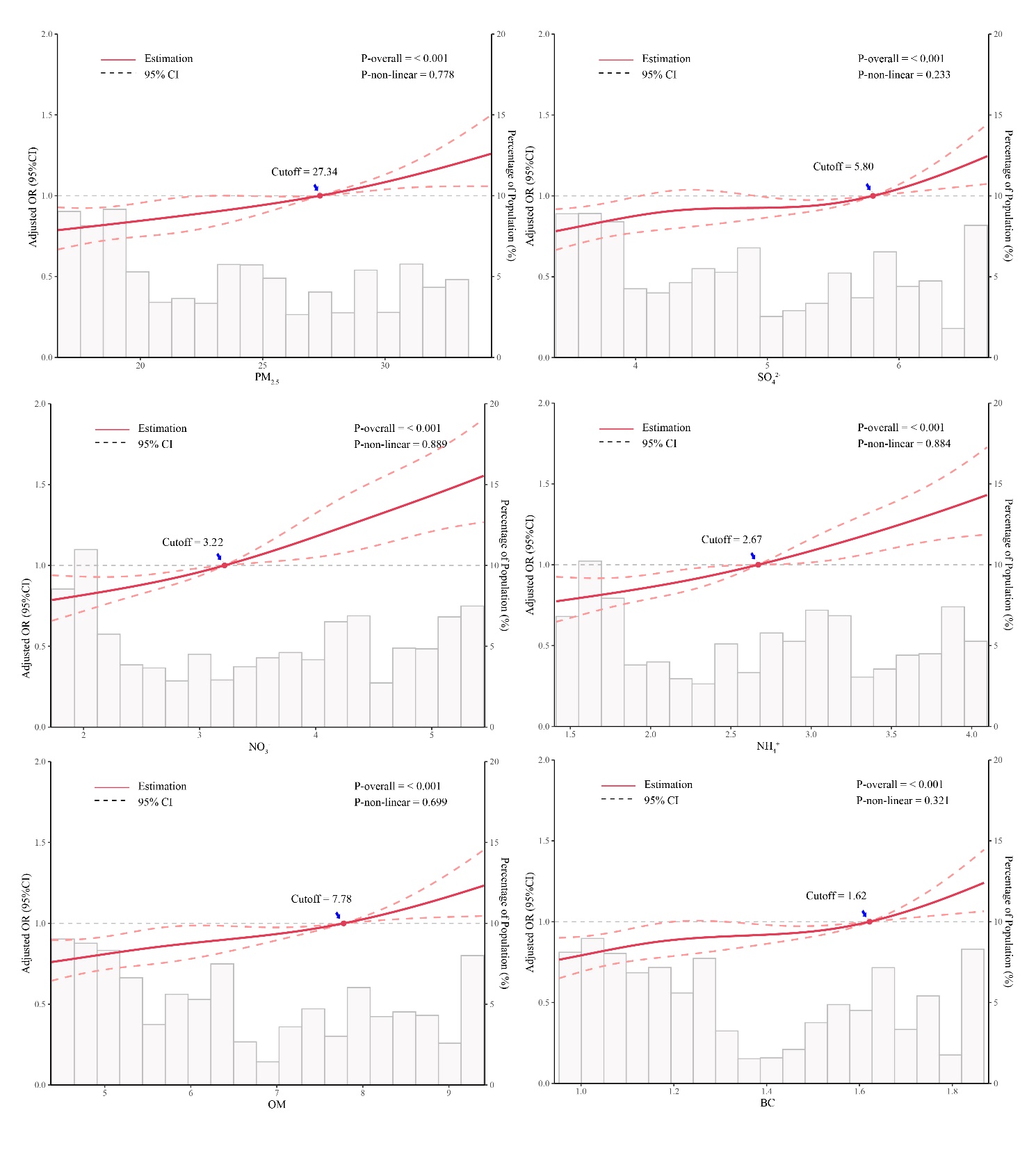


Figure S2 Association between predicted exposure to PM_2.5_ and its constituents during the first to second trimester and GDM risk. The solid line indicates the OR, and the dashed area indicates the 95% CI. The reference point is the lowest value for PM_2.5_ and its constituents, and the nodes are at the 5th, 35th, 65th, and 95th percentiles for PM_2.5_ and its constituents.

## Table S1 Adjusted Correlation Coefficients After PM_2.5_ Outlier Removal

|  | PM_2.5_ | SO_4_^2-^ | NO_3_^-^ | NH_4_^+^ | OM | BC |
| --- | --- | --- | --- | --- | --- | --- |
| PM_2.5_ | 1.000 |  |  |  |  |  |
| SO_4_^2-^ | 0.985 | 1.000 |  |  |  |  |
| NO_3_^-^ | 0.937 | 0.901 | 1.000 |  |  |  |
| NH_4_^+^ | 0.961 | 0.933 | 0.995 | 1.000 |  |  |
| OM | 0.994 | 0.989 | 0.916 | 0.943 | 1.000 |  |
| BC | 0.980 | 0.991 | 0.878 | 0.913 | 0.993 | 1.000 |

*PM_2.5_*, fine particulate matter. *SO_4_^2-^*, sulfate. *NO_3_^-^*, nitrate. *NH_4_^+^*, ammonium. *OM*, organic matter. *BC*, black carbon.

All correlations are significant at *P* <0.001

## Table S2 Subgroup analysis of the association between NO_3_^-^ exposure in the first trimester and GDM risk

| Subgroup | Crude | | |  | Adjusted^a^ | | |
| --- | --- | --- | --- | --- | --- | --- | --- |
|  | OR (95%CI) | *p*-value | *p* for interaction |  | OR (95%CI) | *p*-value | *p* for interaction |
| Age |  |  | 0.123 |  |  |  | 0.074 |
| < 35 years | 0.998 (0.973-1.024) | 0.900 |  |  | 1.217 (1.138-1.301) | < 0.001 |  |
| ≥ 35 years | 0.956 (0.911-1.004) | 0.070 |  |  | 1.049 (0.926-1.188) | 0.452 |  |
| Ethnicity |  |  | 0.404 |  |  |  | 0.434 |
| Han | 0.992 (0.970-1.015) | 0.490 |  |  | 1.172 (1.105-1.244) | < 0.001 |  |
| Other | 0.925 (0.785-1.089) | 0.348 |  |  | 1.076 (0.710-1.631) | 0.730 |  |
| Occupation type |  |  | 0.546 |  |  |  | 0.786 |
| Employee | 0.987 (0.957-1.017) | 0.388 |  |  | 1.168 (1.077-1.266) | < 0.001 |  |
| Freelancer | 0.942 (0.869-1.021) | 0.146 |  |  | 1.113 (0.903-1.371) | 0.317 |  |
| Other | 1.001 (0.966-1.038) | 0.955 |  |  | 1.190 (1.080-1.311) | < 0.001 |  |
| Marital status |  |  | 0.189 |  |  |  | 0.209 |
| Married | 0.993 (0.971-1.016) | 0.553 |  |  | 1.182 (1.113-1.254) | < 0.001 |  |
| Unmarried | 0.904 (0.786-1.039) | 0.154 |  |  | 0.889 (0.611-1.293) | 0.538 |  |
| Blood type |  |  | 0.472 |  |  |  | 0.462 |
| Type A | 0.982 (0.940-1.026) | 0.413 |  |  | 1.206 (1.077-1.351) | < 0.001 |  |
| Type B | 0.970 (0.928-1.014) | 0.176 |  |  | 1.091 (0.971-1.224) | 0.142 |  |
| Type O | 1.002 (0.968-1.038) | 0.898 |  |  | 1.163 (1.060-1.276) | < 0.001 |  |
| Type AB | 1.031 (0.946-1.123) | 0.493 |  |  | 1.458 (1.166-1.823) | < 0.001 |  |
| Nonprimary status |  |  | 0.413 |  |  |  | 0.109 |
| No | 1.004 (0.973-1.036) | 0.800 |  |  | 1.227 (1.137-1.323) | < 0.001 |  |
| Yes | 0.985 (0.954-1.018) | 0.374 |  |  | 1.101 (0.999-1.214) | 0.052 |  |
| Anemia status |  |  | 0.322 |  |  |  | 0.428 |
| No | 1.000 (0.970-1.030) | 0.987 |  |  | 1.178 (1.088-1.275) | < 0.001 |  |
| Yes | 0.977 (0.944-1.011) | 0.184 |  |  | 1.167 (1.069-1.273) | 0.001 |  |
| Infant gender |  |  | 0.124 |  |  |  | 0.106 |
| Male | 0.975 (0.945-1.005) | 0.101 |  |  | 1.135 (1.047-1.230) | 0.002 |  |
| Female | 1.010 (0.977-1.043) | 0.567 |  |  | 1.215 (1.115-1.324) | < 0.001 |  |

^a^ Adjusted for age, ethnicity, occupation type, marital status, blood type, nonprimary status, anaemia status, infant weight, preeclampsia status, vaginitis status, gestational hypertension status, thyroid disease status, temperature, and relative humidity.

## Table S3 Subgroup analysis of the association between NH_4_^+-^ exposure in the first trimester and GDM risk

| Subgroup | Crude | | |  | Adjusted^a^ | | |
| --- | --- | --- | --- | --- | --- | --- | --- |
|  | OR (95%CI) | *p*-value | *p* for interaction |  | OR (95%CI) | *p*-value | *p* for interaction |
| Age |  |  | 0.107 |  |  |  | 0.067 |
| < 35 years | 1.003 (0.966-1.042) | 0.862 |  |  | 1.289 (1.181-1.406) | < 0.001 |  |
| ≥ 35 years | 0.938 (0.873-1.008) | 0.084 |  |  | 1.068 (0.907-1.257) | 0.431 |  |
| Ethnicity |  |  | 0.454 |  |  |  | 0.455 |
| Han | 0.992 (0.960-1.026) | 0.655 |  |  | 1.232 (1.140-1.331) | < 0.001 |  |
| Other | 0.904 (0.711-1.151) | 0.413 |  |  | 1.047 (0.606-1.808) | 0.870 |  |
| Occupation type |  |  | 0.500 |  |  |  | 0.675 |
| Employee | 0.983 (0.939-1.029) | 0.451 |  |  | 1.211 (1.091-1.344) | < 0.001 |  |
| Freelancer | 0.923 (0.819-1.040) | 0.187 |  |  | 1.162 (0.883-1.529) | 0.284 |  |
| Other | 1.007 (0.954-1.062) | 0.808 |  |  | 1.268 (1.117-1.440) | < 0.001 |  |
| Marital status |  |  | 0.157 |  |  |  | 0.177 |
| Married | 0.994 (0.961-1.028) | 0.744 |  |  | 1.244 (1.152-1.344) | < 0.001 |  |
| Unmarried | 0.856 (0.697-1.051) | 0.137 |  |  | 0.837 (0.515-1.358) | 0.470 |  |
| Blood type |  |  | 0.475 |  |  |  | 0.450 |
| Type A | 0.979 (0.917-1.044) | 0.513 |  |  | 1.275 (1.100-1.479) | 0.001 |  |
| Type B | 0.957 (0.896-1.021) | 0.180 |  |  | 1.108 (0.953-1.288) | 0.184 |  |
| Type O | 1.009 (0.958-1.063) | 0.744 |  |  | 1.230 (1.090-1.388) | 0.001 |  |
| Type AB | 1.057 (0.931-1.202) | 0.391 |  |  | 1.618 (1.211-2.163) | 0.001 |  |
| Nonprimary status |  |  | 0.452 |  |  |  | 0.113 |
| No | 1.011 (0.965-1.058) | 0.653 |  |  | 1.297 (1.174-1.432) | < 0.001 |  |
| Yes | 0.985 (0.939-1.034) | 0.542 |  |  | 1.151 (1.015-1.304) | 0.028 |  |
| Anemia status |  |  | 0.335 |  |  |  | 0.412 |
| No | 1.004 (0.961-1.050) | 0.851 |  |  | 1.236 (1.115-1.371) | < 0.001 |  |
| Yes | 0.972 (0.924-1.022) | 0.263 |  |  | 1.227 (1.095-1.374) | < 0.001 |  |
| Infant gender |  |  | 0.177 |  |  |  | 0.149 |
| Male | 0.97 (0.927-1.015) | 0.186 |  |  | 1.198 (1.079-1.330) | 0.001 |  |
| Female | 1.015 (0.967-1.066) | 0.541 |  |  | 1.267 (1.134-1.417) | < 0.001 |  |

^a^ Adjusted for age, ethnicity, occupation type, marital status, blood type, nonprimary status, anaemia status, infant weight, preeclampsia status, vaginitis status, gestational hypertension status, thyroid disease status, temperature, and relative humidity.

## Table S4 Subgroup analysis of the association between OM exposure in the first trimester and GDM risk

| Subgroup | Crude | | |  | Adjusted^a^ | | |
| --- | --- | --- | --- | --- | --- | --- | --- |
|  | OR (95%CI) | *p*-value | *p* for interaction |  | OR (95%CI) | *p*-value | *p* for interaction |
| Age |  |  | 0.073 |  |  |  | 0.046 |
| < 35 years | 1.019 (0.997-1.042) | 0.093 |  |  | 1.114 (1.076-1.153) | < 0.001 |  |
| ≥ 35 years | 0.975 (0.935-1.018) | 0.251 |  |  | 1.038 (0.974-1.107) | 0.247 |  |
| Ethnicity |  |  | 0.742 |  |  |  | 0.722 |
| Han | 1.009 (0.989-1.029) | 0.371 |  |  | 1.096 (1.063-1.130) | < 0.001 |  |
| Other | 0.985 (0.854-1.136) | 0.834 |  |  | 0.991 (0.794-1.236) | 0.934 |  |
| Occupation type |  |  | 0.427 |  |  |  | 0.576 |
| Employee | 1.001 (0.974-1.028) | 0.965 |  |  | 1.085 (1.042-1.130) | < 0.001 |  |
| Freelancer | 0.978 (0.914-1.048) | 0.532 |  |  | 1.080 (0.966-1.208) | 0.177 |  |
| Other | 1.017 (0.987-1.049) | 0.269 |  |  | 1.110 (1.055-1.169) | < 0.001 |  |
| Marital status |  |  | 0.081 |  |  |  | 0.099 |
| Married | 1.011 (0.991-1.031) | 0.272 |  |  | 1.100 (1.067-1.135) | < 0.001 |  |
| Unmarried | 0.904 (0.798-1.024) | 0.111 |  |  | 0.903 (0.745-1.095) | 0.300 |  |
| Blood type |  |  | 0.637 |  |  |  | 0.603 |
| Type A | 1.007 (0.969-1.045) | 0.734 |  |  | 1.110 (1.047-1.177) | < 0.001 |  |
| Type B | 0.980 (0.944-1.018) | 0.305 |  |  | 1.041 (0.981-1.106) | 0.185 |  |
| Type O | 1.018 (0.988-1.050) | 0.244 |  |  | 1.102 (1.050-1.155) | < 0.001 |  |
| Type AB | 1.066 (0.990-1.149) | 0.091 |  |  | 1.210 (1.079-1.356) | 0.001 |  |
| Nonprimary status |  |  | 0.584 |  |  |  | 0.140 |
| No | 1.022 (0.995-1.050) | 0.106 |  |  | 1.110 (1.066-1.155) | < 0.001 |  |
| Yes | 1.011 (0.982-1.041) | 0.456 |  |  | 1.079 (1.029-1.131) | 0.002 |  |
| Anemia status |  |  | 0.247 |  |  |  | 0.261 |
| No | 1.020 (0.994-1.047) | 0.131 |  |  | 1.103 (1.059-1.150) | < 0.001 |  |
| Yes | 0.997 (0.968-1.027) | 0.834 |  |  | 1.085 (1.037-1.135) | < 0.001 |  |
| Infant gender |  |  | 0.318 |  |  |  | 0.246 |
| Male | 0.999 (0.973-1.026) | 0.961 |  |  | 1.087 (1.043-1.133) | < 0.001 |  |
| Female | 1.019 (0.991-1.049) | 0.187 |  |  | 1.103 (1.055-1.153) | < 0.001 |  |

^a^ Adjusted for age, ethnicity, occupation type, marital status, blood type, nonprimary status, anaemia status, infant weight, preeclampsia status, vaginitis status, gestational hypertension status, thyroid disease status, temperature, and relative humidity.

## Table S5 Subgroup analysis of the association between BC exposure in the first trimester and GDM risk

| Subgroup | Crude | | |  | Adjusted^a^ | | |
| --- | --- | --- | --- | --- | --- | --- | --- |
|  | OR (95%CI) | *p*-value | *p* for interaction |  | OR (95%CI) | *p*-value | *p* for interaction |
| Age |  |  | 0.067 |  |  |  | 0.044 |
| < 35 years | 1.163 (1.029-1.315) | 0.015 |  |  | 1.632 (1.381-1.928) | < 0.001 |  |
| ≥ 35 years | 0.908 (0.717-1.149) | 0.420 |  |  | 1.182 (0.868-1.609) | 0.290 |  |
| Ethnicity |  |  | 0.958 |  |  |  | 0.897 |
| Han | 1.095 (0.982-1.221) | 0.103 |  |  | 1.516 (1.308-1.757) | < 0.001 |  |
| Other | 1.072 (0.488-2.355) | 0.863 |  |  | 1.012 (0.343-2.992) | 0.982 |  |
| Occupation type |  |  | 0.445 |  |  |  | 0.484 |
| Employee | 1.048 (0.901-1.220) | 0.539 |  |  | 1.439 (1.185-1.747) | < 0.001 |  |
| Freelancer | 0.927 (0.632-1.360) | 0.698 |  |  | 1.415 (0.816-2.453) | 0.216 |  |
| Other | 1.146 (0.966-1.361) | 0.119 |  |  | 1.624 (1.266-2.083) | < 0.001 |  |
| Marital status |  |  | 0.063 |  |  |  | 0.080 |
| Married | 1.111 (0.996-1.239) | 0.060 |  |  | 1.545 (1.332-1.792) | < 0.001 |  |
| Unmarried | 0.574 (0.289-1.141) | 0.113 |  |  | 0.612 (0.244-1.538) | 0.296 |  |
| Blood type |  |  | 0.661 |  |  |  | 0.632 |
| Type A | 1.092 (0.887-1.344) | 0.406 |  |  | 1.614 (1.216-2.143) | 0.001 |  |
| Type B | 0.918 (0.743-1.134) | 0.426 |  |  | 1.159 (0.868-1.549) | 0.318 |  |
| Type O | 1.160 (0.978-1.375) | 0.088 |  |  | 1.583 (1.258-1.992) | < 0.001 |  |
| Type AB | 1.513 (0.999-2.292) | 0.051 |  |  | 2.360 (1.353-4.117) | 0.002 |  |
| Nonprimary status |  |  | 0.705 |  |  |  | 0.135 |
| No | 1.180 (1.015-1.370) | 0.031 |  |  | 1.617 (1.326-1.971) | < 0.001 |  |
| Yes | 1.131 (0.963-1.327) | 0.134 |  |  | 1.402 (1.122-1.752) | 0.003 |  |
| Anemia status |  |  | 0.283 |  |  |  | 0.300 |
| No | 1.165 (1.008-1.346) | 0.039 |  |  | 1.557 (1.278-1.896) | < 0.001 |  |
| Yes | 1.034 (0.878-1.217) | 0.689 |  |  | 1.454 (1.169-1.808) | 0.001 |  |
| Infant gender |  |  | 0.400 |  |  |  | 0.297 |
| Male | 1.048 (0.905-1.215) | 0.531 |  |  | 1.464 (1.198-1.788) | < 0.001 |  |
| Female | 1.150 (0.982-1.348) | 0.082 |  |  | 1.556 (1.256-1.928) | < 0.001 |  |

^a^ Adjusted for age, ethnicity, occupation type, marital status, blood type, nonprimary status, anaemia status, infant weight, preeclampsia status, vaginitis status, gestational hypertension status, thyroid disease status, temperature, and relative humidity.

## Table S6 Subgroup analysis of the association between PM_2.5_ exposure in the second trimester and GDM risk

| Subgroup | Crude | | |  | Adjusted^a^ | | |
| --- | --- | --- | --- | --- | --- | --- | --- |
|  | OR (95%CI) | *p*-value | *p* for interaction |  | OR (95%CI) | *p*-value | *p* for interaction |
| Age |  |  | 0.754 |  |  |  | 0.708 |
| < 35 years | 1.001 (0.995-1.006) | 0.838 |  |  | 1.018 (1.006-1.029) | 0.003 |  |
| ≥ 35 years | 1.002 (0.992-1.013) | 0.649 |  |  | 1.020 (0.998-1.042) | 0.076 |  |
| Ethnicity |  |  | 0.407 |  |  |  | 0.422 |
| Han | 1.000 (0.995-1.005) | 0.896 |  |  | 1.018 (1.007-1.028) | 0.001 |  |
| Other | 1.013 (0.982-1.046) | 0.413 |  |  | 1.010 (0.943-1.083) | 0.769 |  |
| Occupation type |  |  | 0.526 |  |  |  | 0.561 |
| Employee | 0.997 (0.991-1.003) | 0.329 |  |  | 1.013 (0.999-1.026) | 0.067 |  |
| Freelancer | 1.021 (1.004-1.039) | 0.017 |  |  | 1.031 (0.992-1.071) | 0.126 |  |
| Other | 1.000 (0.992-1.008) | 0.973 |  |  | 1.023 (1.006-1.040) | 0.007 |  |
| Marital status |  |  | 0.483 |  |  |  | 0.538 |
| Married | 1.000 (0.995-1.005) | 0.907 |  |  | 1.019 (1.009-1.029) | < 0.001 |  |
| Unmarried | 1.011 (0.980-1.042) | 0.489 |  |  | 0.980 (0.918-1.046) | 0.546 |  |
| Blood type |  |  | 0.223 |  |  |  | 0.265 |
| Type A | 1.004 (0.995-1.013) | 0.401 |  |  | 1.030 (1.010-1.050) | 0.003 |  |
| Type B | 0.999 (0.990-1.008) | 0.821 |  |  | 1.009 (0.989-1.030) | 0.374 |  |
| Type O | 0.997 (0.989-1.004) | 0.367 |  |  | 1.012 (0.996-1.028) | 0.135 |  |
| Type AB | 1.008 (0.990-1.026) | 0.399 |  |  | 1.032 (0.992-1.073) | 0.114 |  |
| Nonprimary status |  |  | 0.522 |  |  |  | 0.568 |
| No | 1.003 (0.996-1.009) | 0.440 |  |  | 1.018 (1.006-1.032) | 0.005 |  |
| Yes | 0.999 (0.992-1.007) | 0.859 |  |  | 1.016 (1.000-1.033) | 0.057 |  |
| Anemia status |  |  | 0.861 |  |  |  | 0.960 |
| No | 1.001 (0.994-1.007) | 0.863 |  |  | 1.014 (1.000-1.028) | 0.057 |  |
| Yes | 1.001 (0.994-1.009) | 0.695 |  |  | 1.022 (1.007-1.037) | 0.003 |  |
| Infant gender |  |  | 0.482 |  |  |  | 0.513 |
| Male | 1.002 (0.995-1.008) | 0.633 |  |  | 1.017 (1.003-1.031) | 0.020 |  |
| Female | 0.998 (0.991-1.005) | 0.606 |  |  | 1.018 (1.004-1.033) | 0.014 |  |

^a^ Adjusted for age, ethnicity, occupation type, marital status, blood type, nonprimary status, anaemia status, infant weight, preeclampsia status, vaginitis status, gestational hypertension status, thyroid disease status, temperature, and relative humidity.

## Table S7 Subgroup analysis of the association between SO_4_^2-^ exposure in the second trimester and GDM risk

| Subgroup | Crude | | |  | Adjusted^a^ | | |
| --- | --- | --- | --- | --- | --- | --- | --- |
|  | OR (95%CI) | *p*-value | *p* for interaction |  | OR (95%CI) | *p*-value | *p* for interaction |
| Age |  |  | 0.741 |  |  |  | 0.678 |
| < 35 years | 1.002 (0.972-1.033) | 0.908 |  |  | 1.046 (0.997-1.097) | 0.069 |  |
| ≥ 35 years | 1.013 (0.954-1.076) | 0.669 |  |  | 1.068 (0.974-1.171) | 0.162 |  |
| Ethnicity |  |  | 0.467 |  |  |  | 0.455 |
| Han | 0.998 (0.971-1.025) | 0.869 |  |  | 1.048 (1.004-1.095) | 0.031 |  |
| Other | 1.066 (0.894-1.270) | 0.477 |  |  | 1.023 (0.762-1.373) | 0.879 |  |
| Occupation type |  |  | 0.422 |  |  |  | 0.515 |
| Employee | 0.981 (0.947-1.016) | 0.285 |  |  | 1.029 (0.972-1.089) | 0.331 |  |
| Freelancer | 1.115 (1.010-1.231) | 0.032 |  |  | 1.104 (0.935-1.304) | 0.245 |  |
| Other | 1.004 (0.960-1.050) | 0.861 |  |  | 1.071 (0.998-1.148) | 0.056 |  |
| Marital status |  |  | 0.614 |  |  |  | 0.709 |
| Married | 0.998 (0.971-1.025) | 0.888 |  |  | 1.052 (1.008-1.099) | 0.020 |  |
| Unmarried | 1.045 (0.876-1.246) | 0.625 |  |  | 0.931 (0.705-1.230) | 0.614 |  |
| Blood type |  |  | 0.146 |  |  |  | 0.178 |
| Type A | 1.027 (0.975-1.082) | 0.309 |  |  | 1.111 (1.024-1.205) | 0.011 |  |
| Type B | 0.992 (0.941-1.045) | 0.757 |  |  | 1.018 (0.935-1.108) | 0.682 |  |
| Type O | 0.978 (0.938-1.019) | 0.291 |  |  | 1.022 (0.956-1.092) | 0.527 |  |
| Type AB | 1.043 (0.943-1.153) | 0.412 |  |  | 1.088 (0.921-1.286) | 0.320 |  |
| Nonprimary status |  |  | 0.538 |  |  |  | 0.361 |
| No | 1.014 (0.979-1.050) | 0.448 |  |  | 1.059 (1.003-1.119) | 0.040 |  |
| Yes | 0.997 (0.956-1.039) | 0.869 |  |  | 1.032 (0.964-1.104) | 0.367 |  |
| Anemia status |  |  | 0.606 |  |  |  | 0.758 |
| No | 0.998 (0.963-1.035) | 0.929 |  |  | 1.023 (0.964-1.085) | 0.457 |  |
| Yes | 1.013 (0.973-1.054) | 0.538 |  |  | 1.076 (1.012-1.144) | 0.019 |  |
| Infant gender |  |  | 0.746 |  |  |  | 0.810 |
| Male | 1.003 (0.967-1.041) | 0.857 |  |  | 1.034 (0.975-1.097) | 0.261 |  |
| Female | 0.995 (0.957-1.034) | 0.784 |  |  | 1.064 (1.000-1.131) | 0.049 |  |

^a^ Adjusted for age, ethnicity, occupation type, marital status, blood type, nonprimary status, anaemia status, infant weight, preeclampsia status, vaginitis status, gestational hypertension status, thyroid disease status, temperature, and relative humidity.

## Table S8 Subgroup analysis of the association between NO_3_^-^ exposure in the second trimester and GDM risk

| Subgroup | Crude | | |  | Adjusted^a^ | | |
| --- | --- | --- | --- | --- | --- | --- | --- |
|  | OR (95%CI) | *p*-value | *p* for interaction |  | OR (95%CI) | *p*-value | *p* for interaction |
| Age |  |  | 0.837 |  |  |  | 0.768 |
| < 35 years | 1.001 (0.977-1.025) | 0.938 |  |  | 1.196 (1.108-1.291) | < 0.001 |  |
| ≥ 35 years | 1.007 (0.960-1.056) | 0.787 |  |  | 1.202 (1.040-1.388) | 0.013 |  |
| Ethnicity |  |  | 0.493 |  |  |  | 0.534 |
| Han | 0.997 (0.976-1.019) | 0.775 |  |  | 1.194 (1.115-1.277) | < 0.001 |  |
| Other | 1.048 (0.910-1.207) | 0.516 |  |  | 1.114 (0.691-1.797) | 0.657 |  |
| Occupation type |  |  | 0.772 |  |  |  | 0.754 |
| Employee | 0.986 (0.959-1.015) | 0.342 |  |  | 1.152 (1.054-1.261) | 0.002 |  |
| Freelancer | 1.109 (1.027-1.198) | 0.008 |  |  | 1.355 (1.054-1.740) | 0.018 |  |
| Other | 0.993 (0.958-1.029) | 0.697 |  |  | 1.221 (1.091-1.366) | < 0.001 |  |
| Marital status |  |  | 0.484 |  |  |  | 0.503 |
| Married | 0.997 (0.976-1.019) | 0.782 |  |  | 1.204 (1.125-1.289) | < 0.001 |  |
| Unmarried | 1.046 (0.916-1.193) | 0.507 |  |  | 0.888 (0.580-1.360) | 0.585 |  |
| Blood type |  |  | 0.377 |  |  |  | 0.406 |
| Type A | 1.008 (0.967-1.050) | 0.720 |  |  | 1.274 (1.119-1.451) | < 0.001 |  |
| Type B | 0.996 (0.955-1.038) | 0.848 |  |  | 1.144 (0.999-1.310) | 0.052 |  |
| Type O | 0.984 (0.952-1.017) | 0.334 |  |  | 1.141 (1.027-1.268) | 0.014 |  |
| Type AB | 1.052 (0.970-1.142) | 0.219 |  |  | 1.397 (1.082-1.804) | 0.010 |  |
| Nonprimary status |  |  | 0.593 |  |  |  | 0.902 |
| No | 1.007 (0.979-1.036) | 0.636 |  |  | 1.157 (1.062-1.260) | 0.001 |  |
| Yes | 0.995 (0.964-1.028) | 0.769 |  |  | 1.266 (1.130-1.417) | < 0.001 |  |
| Anemia status |  |  | 0.700 |  |  |  | 0.501 |
| No | 1.006 (0.977-1.035) | 0.703 |  |  | 1.189 (1.083-1.305) | < 0.001 |  |
| Yes | 0.997 (0.966-1.030) | 0.862 |  |  | 1.201 (1.090-1.323) | < 0.001 |  |
| Infant gender |  |  | 0.303 |  |  |  | 0.293 |
| Male | 1.008 (0.980-1.038) | 0.571 |  |  | 1.227 (1.119-1.347) | < 0.001 |  |
| Female | 0.986 (0.956-1.017) | 0.379 |  |  | 1.155 (1.048-1.273) | 0.004 |  |

^a^ Adjusted for age, ethnicity, occupation type, marital status, blood type, nonprimary status, anaemia status, infant weight, preeclampsia status, vaginitis status, gestational hypertension status, thyroid disease status, temperature, and relative humidity.

## Table S9 Subgroup analysis of the association between NH_4_^+^ exposure in the second trimester and GDM risk

| Subgroup | Crude | | |  | Adjusted^a^ | | |
| --- | --- | --- | --- | --- | --- | --- | --- |
|  | OR (95%CI) | *p*-value | *p* for interaction |  | OR (95%CI) | *p*-value | *p* for interaction |
| Age |  |  | 0.766 |  |  |  | 0.706 |
| < 35 years | 1.002 (0.967-1.038) | 0.923 |  |  | 1.203 (1.094-1.322) | < 0.001 |  |
| ≥ 35 years | 1.014 (0.945-1.087) | 0.703 |  |  | 1.243 (1.040-1.486) | 0.017 |  |
| Ethnicity |  |  | 0.489 |  |  |  | 0.509 |
| Han | 0.997 (0.966-1.028) | 0.828 |  |  | 1.207 (1.109-1.312) | < 0.001 |  |
| Other | 1.073 (0.873-1.319) | 0.505 |  |  | 1.102 (0.612-1.986) | 0.746 |  |
| Occupation type |  |  | 0.701 |  |  |  | 0.697 |
| Employee | 0.980 (0.940-1.022) | 0.344 |  |  | 1.158 (1.036-1.294) | 0.010 |  |
| Freelancer | 1.163 (1.039-1.303) | 0.009 |  |  | 1.416 (1.030-1.945) | 0.032 |  |
| Other | 0.993 (0.943-1.046) | 0.792 |  |  | 1.242 (1.082-1.425) | 0.002 |  |
| Marital status |  |  | 0.475 |  |  |  | 0.499 |
| Married | 0.997 (0.966-1.028) | 0.830 |  |  | 1.218 (1.119-1.325) | < 0.001 |  |
| Unmarried | 1.071 (0.881-1.303) | 0.491 |  |  | 0.886 (0.516-1.519) | 0.659 |  |
| Blood type |  |  | 0.299 |  |  |  | 0.338 |
| Type A | 1.017 (0.957-1.081) | 0.588 |  |  | 1.324 (1.129-1.552) | 0.001 |  |
| Type B | 0.994 (0.935-1.057) | 0.847 |  |  | 1.142 (0.965-1.351) | 0.122 |  |
| Type O | 0.976 (0.930-1.025) | 0.325 |  |  | 1.139 (1.000-1.297) | 0.050 |  |
| Type AB | 1.074 (0.953-1.209) | 0.242 |  |  | 1.430 (1.039-1.967) | 0.028 |  |
| Nonprimary status |  |  | 0.602 |  |  |  | 0.913 |
| No | 1.012 (0.97-1.055) | 0.581 |  |  | 1.179 (1.060-1.310) | 0.002 |  |
| Yes | 0.995 (0.949-1.043) | 0.834 |  |  | 1.263 (1.099-1.451) | 0.001 |  |
| Anemia status |  |  | 0.883 |  |  |  | 0.674 |
| No | 1.006 (0.965-1.049) | 0.772 |  |  | 1.178 (1.049-1.323) | 0.006 |  |
| Yes | 1.001 (0.956-1.049) | 0.950 |  |  | 1.238 (1.099-1.394) | < 0.001 |  |
| Infant gender |  |  | 0.369 |  |  |  | 0.361 |
| Male | 1.012 (0.969-1.056) | 0.596 |  |  | 1.225 (1.092-1.374) | 0.001 |  |
| Female | 0.983 (0.939-1.029) | 0.462 |  |  | 1.184 (1.050-1.336) | 0.006 |  |

^a^ Adjusted for age, ethnicity, occupation type, marital status, blood type, nonprimary status, anaemia status, infant weight, preeclampsia status, vaginitis status, gestational hypertension status, thyroid disease status, temperature, and relative humidity.

## Table S10 Subgroup analysis of the association between OM exposure in the second trimester and GDM risk

| Subgroup | Crude | | |  | Adjusted^a^ | | |
| --- | --- | --- | --- | --- | --- | --- | --- |
|  | OR (95%CI) | *p*-value | *p* for interaction |  | OR (95%CI) | *p*-value | *p* for interaction |
| Age |  |  | 0.745 |  |  |  | 0.689 |
| < 35 years | 1.004 (0.984-1.024) | 0.710 |  |  | 1.052 (1.014-1.091) | 0.007 |  |
| ≥ 35 years | 1.011 (0.972-1.052) | 0.581 |  |  | 1.062 (0.990-1.138) | 0.092 |  |
| Ethnicity |  |  | 0.439 |  |  |  | 0.460 |
| Han | 1.001 (0.983-1.019) | 0.944 |  |  | 1.053 (1.019-1.087) | 0.002 |  |
| Other | 1.048 (0.933-1.178) | 0.428 |  |  | 1.000 (0.799-1.252) | 0.998 |  |
| Occupation type |  |  | 0.438 |  |  |  | 0.500 |
| Employee | 0.989 (0.966-1.013) | 0.377 |  |  | 1.036 (0.992-1.081) | 0.113 |  |
| Freelancer | 1.074 (1.007-1.144) | 0.029 |  |  | 1.078 (0.951-1.222) | 0.240 |  |
| Other | 1.004 (0.975-1.034) | 0.775 |  |  | 1.073 (1.018-1.132) | 0.009 |  |
| Marital status |  |  | 0.637 |  |  |  | 0.704 |
| Married | 1.001 (0.983-1.019) | 0.917 |  |  | 1.056 (1.022-1.091) | 0.001 |  |
| Unmarried | 1.030 (0.917-1.156) | 0.621 |  |  | 0.919 (0.744-1.135) | 0.433 |  |
| Blood type |  |  | 0.154 |  |  |  | 0.193 |
| Type A | 1.020 (0.986-1.056) | 0.258 |  |  | 1.100 (1.034-1.170) | 0.003 |  |
| Type B | 0.996 (0.962-1.032) | 0.838 |  |  | 1.022 (0.958-1.091) | 0.503 |  |
| Type O | 0.988 (0.961-1.016) | 0.387 |  |  | 1.032 (0.981-1.085) | 0.227 |  |
| Type AB | 1.030 (0.963-1.101) | 0.388 |  |  | 1.104 (0.973-1.252) | 0.125 |  |
| Nonprimary status |  |  | 0.581 |  |  |  | 0.491 |
| No | 1.011 (0.988-1.035) | 0.356 |  |  | 1.058 (1.015-1.103) | 0.007 |  |
| Yes | 1.001 (0.973-1.029) | 0.959 |  |  | 1.041 (0.988-1.097) | 0.132 |  |
| Anemia status |  |  | 0.749 |  |  |  | 0.919 |
| No | 1.003 (0.979-1.027) | 0.819 |  |  | 1.037 (0.992-1.085) | 0.108 |  |
| Yes | 1.009 (0.982-1.036) | 0.522 |  |  | 1.069 (1.020-1.120) | 0.005 |  |
| Infant gender |  |  | 0.574 |  |  |  | 0.648 |
| Male | 1.006 (0.982-1.031) | 0.601 |  |  | 1.045 (0.999-1.093) | 0.053 |  |
| Female | 0.996 (0.971-1.022) | 0.779 |  |  | 1.059 (1.011-1.110) | 0.015 |  |

^a^ Adjusted for age, ethnicity, occupation type, marital status, blood type, nonprimary status, anaemia status, infant weight, preeclampsia status, vaginitis status, gestational hypertension status, thyroid disease status, temperature, and relative humidity.

## Table S11 Subgroup analysis of the association between BC exposure in the second trimester and GDM risk

| Subgroup | Crude | | |  | Adjusted^a^ | | |
| --- | --- | --- | --- | --- | --- | --- | --- |
|  | OR (95%CI) | *p*-value | *p* for interaction |  | OR (95%CI) | *p*-value | *p* for interaction |
| Age |  |  | 0.735 |  |  |  | 0.668 |
| < 35 years | 1.015 (0.909-1.133) | 0.795 |  |  | 1.186 (0.997-1.411) | 0.054 |  |
| ≥ 35 years | 1.059 (0.850-1.319) | 0.610 |  |  | 1.270 (0.911-1.771) | 0.159 |  |
| Ethnicity |  |  | 0.491 |  |  |  | 0.507 |
| Han | 0.999 (0.905-1.103) | 0.984 |  |  | 1.199 (1.026-1.400) | 0.022 |  |
| Other | 1.255 (0.660-2.388) | 0.488 |  |  | 0.906 (0.309-2.656) | 0.858 |  |
| Occupation type |  |  | 0.373 |  |  |  | 0.444 |
| Employee | 0.936 (0.821-1.067) | 0.324 |  |  | 1.110 (0.905-1.362) | 0.315 |  |
| Freelancer | 1.417 (0.993-2.022) | 0.055 |  |  | 1.285 (0.702-2.352) | 0.417 |  |
| Other | 1.030 (0.875-1.212) | 0.726 |  |  | 1.326 (1.027-1.712) | 0.030 |  |
| Marital status |  |  | 0.716 |  |  |  | 0.813 |
| Married | 1.001 (0.906-1.105) | 0.991 |  |  | 1.215 (1.040-1.420) | 0.014 |  |
| Unmarried | 1.129 (0.593-2.150) | 0.712 |  |  | 0.705 (0.263-1.889) | 0.486 |  |
| Blood type |  |  | 0.124 |  |  |  | 0.155 |
| Type A | 1.123 (0.928-1.360) | 0.234 |  |  | 1.488 (1.108-1.999) | 0.008 |  |
| Type B | 0.972 (0.803-1.178) | 0.773 |  |  | 1.051 (0.774-1.427) | 0.750 |  |
| Type O | 0.927 (0.795-1.080) | 0.330 |  |  | 1.089 (0.856-1.385) | 0.489 |  |
| Type AB | 1.155 (0.800-1.668) | 0.442 |  |  | 1.425 (0.779-2.608) | 0.250 |  |
| Nonprimary status |  |  | 0.564 |  |  |  | 0.336 |
| No | 1.059 (0.932-1.205) | 0.380 |  |  | 1.252 (1.024-1.532) | 0.029 |  |
| Yes | 0.998 (0.856-1.165) | 0.985 |  |  | 1.110 (0.873-1.413) | 0.394 |  |
| Anemia status |  |  | 0.623 |  |  |  | 0.775 |
| No | 1.002 (0.878-1.144) | 0.972 |  |  | 1.106 (0.895-1.366) | 0.353 |  |
| Yes | 1.053 (0.910-1.219) | 0.487 |  |  | 1.304 (1.043-1.631) | 0.020 |  |
| Infant gender |  |  | 0.692 |  |  |  | 0.800 |
| Male | 1.023 (0.894-1.170) | 0.739 |  |  | 1.142 (0.924-1.412) | 0.219 |  |
| Female | 0.983 (0.853-1.134) | 0.818 |  |  | 1.255 (1.004-1.568) | 0.046 |  |

^a^ Adjusted for age, ethnicity, occupation type, marital status, blood type, nonprimary status, anaemia status, infant weight, preeclampsia status, vaginitis status, gestational hypertension status, thyroid disease status, temperature, and relative humidity.

## Table S12 Subgroup analysis of the association between PM_2.5_ exposure in the first to second trimester and GDM risk

| Subgroup | Crude | | |  | Adjusted^a^ | | |
| --- | --- | --- | --- | --- | --- | --- | --- |
|  | OR (95%CI) | *p*-value | *p* for interaction |  | OR (95%CI) | *p*-value | *p* for interaction |
| Age |  |  | 0.364 |  |  |  | 0.296 |
| < 35 years | 1.003 (0.996-1.011) | 0.379 |  |  | 1.035 (1.023-1.047) | < 0.001 |  |
| ≥ 35 years | 0.996 (0.981-1.011) | 0.566 |  |  | 1.020 (0.998-1.043) | 0.072 |  |
| Ethnicity |  |  | 0.730 |  |  |  | 0.740 |
| Han | 1.001 (0.994-1.007) | 0.86 |  |  | 1.031 (1.020-1.042) | < 0.001 |  |
| Other | 1.009 (0.964-1.055) | 0.708 |  |  | 1.009 (0.941-1.083) | 0.794 |  |
| Occupation type |  |  | 0.342 |  |  |  | 0.529 |
| Employee | 0.996 (0.987-1.005) | 0.411 |  |  | 1.027 (1.013-1.041) | < 0.001 |  |
| Freelancer | 1.013 (0.989-1.038) | 0.281 |  |  | 1.042 (1.002-1.083) | 0.040 |  |
| Other | 1.003 (0.992-1.014) | 0.582 |  |  | 1.034 (1.017-1.052) | < 0.001 |  |
| Marital status |  |  | 0.569 |  |  |  | 0.557 |
| Married | 1.001 (0.994-1.008) | 0.766 |  |  | 1.032 (1.022-1.043) | < 0.001 |  |
| Unmarried | 0.989 (0.947-1.032) | 0.596 |  |  | 0.974 (0.909-1.044) | 0.457 |  |
| Blood type |  |  | 0.555 |  |  |  | 0.618 |
| Type A | 1.004 (0.991-1.018) | 0.503 |  |  | 1.040 (1.020-1.061) | < 0.001 |  |
| Type B | 0.994 (0.981-1.007) | 0.364 |  |  | 1.019 (0.997-1.040) | 0.085 |  |
| Type O | 0.999 (0.989-1.010) | 0.913 |  |  | 1.027 (1.010-1.044) | 0.001 |  |
| Type AB | 1.018 (0.993-1.044) | 0.156 |  |  | 1.062 (1.021-1.104) | 0.002 |  |
| Nonprimary status |  |  | 0.406 |  |  |  | 0.435 |
| No | 1.006 (0.997-1.015) | 0.193 |  |  | 1.031 (1.018-1.044) | < 0.001 |  |
| Yes | 1.000 (0.990-1.011) | 0.971 |  |  | 1.032 (1.014-1.051) | 0.001 |  |
| Anemia status |  |  | 0.659 |  |  |  | 0.441 |
| No | 1.003 (0.994-1.012) | 0.468 |  |  | 1.031 (1.016-1.046) | < 0.001 |  |
| Yes | 1.000 (0.990-1.010) | 0.958 |  |  | 1.031 (1.016-1.047) | < 0.001 |  |
| Infant gender |  |  | 0.819 |  |  |  | 0.705 |
| Male | 1.000 (0.991-1.009) | 0.990 |  |  | 1.030 (1.016-1.045) | < 0.001 |  |
| Female | 1.002 (0.992-1.011) | 0.745 |  |  | 1.031 (1.016-1.047) | < 0.001 |  |

^a^ Adjusted for age, ethnicity, occupation type, marital status, blood type, nonprimary status, anaemia status, infant weight, preeclampsia status, vaginitis status, gestational hypertension status, thyroid disease status, temperature, and relative humidity.

## Table S13 Subgroup analysis of the association between SO_4_^2-^ exposure in the first to second trimester and GDM risk

| Subgroup | Crude | | |  | Adjusted^a^ | | |
| --- | --- | --- | --- | --- | --- | --- | --- |
|  | OR (95%CI) | *p*-value | *p* for interaction |  | OR (95%CI) | *p*-value | *p* for interaction |
| Age |  |  | 0.340 |  |  |  | 0.269 |
| < 35 years | 1.029 (0.987-1.074) | 0.177 |  |  | 1.164 (1.099-1.234) | < 0.001 |  |
| ≥ 35 years | 0.984 (0.905-1.069) | 0.698 |  |  | 1.090 (0.978-1.216) | 0.121 |  |
| Ethnicity |  |  | 0.678 |  |  |  | 0.716 |
| Han | 1.013 (0.976-1.052) | 0.501 |  |  | 1.145 (1.087-1.205) | < 0.001 |  |
| Other | 1.069 (0.831-1.375) | 0.603 |  |  | 1.027 (0.723-1.459) | 0.882 |  |
| Occupation type |  |  | 0.255 |  |  |  | 0.424 |
| Employee | 0.985 (0.936-1.037) | 0.566 |  |  | 1.117 (1.043-1.196) | 0.002 |  |
| Freelancer | 1.082 (0.947-1.236) | 0.246 |  |  | 1.211 (0.998-1.470) | 0.053 |  |
| Other | 1.031 (0.971-1.096) | 0.315 |  |  | 1.168 (1.073-1.272) | < 0.001 |  |
| Marital status |  |  | 0.379 |  |  |  | 0.370 |
| Married | 1.016 (0.979-1.055) | 0.398 |  |  | 1.152 (1.094-1.214) | < 0.001 |  |
| Unmarried | 0.912 (0.717-1.158) | 0.449 |  |  | 0.854 (0.607-1.201) | 0.364 |  |
| Blood type |  |  | 0.487 |  |  |  | 0.547 |
| Type A | 1.038 (0.966-1.116) | 0.311 |  |  | 1.198 (1.086-1.321) | < 0.001 |  |
| Type B | 0.974 (0.906-1.048) | 0.485 |  |  | 1.077 (0.972-1.194) | 0.157 |  |
| Type O | 1.004 (0.947-1.065) | 0.881 |  |  | 1.121 (1.035-1.215) | 0.005 |  |
| Type AB | 1.125 (0.979-1.293) | 0.097 |  |  | 1.326 (1.092-1.609) | 0.004 |  |
| Nonprimary status |  |  | 0.628 |  |  |  | 0.445 |
| No | 1.041 (0.991-1.094) | 0.113 |  |  | 1.147 (1.076-1.222) | < 0.001 |  |
| Yes | 1.021 (0.963-1.083) | 0.482 |  |  | 1.145 (1.049-1.251) | 0.003 |  |
| Anemia status |  |  | 0.848 |  |  |  | 0.597 |
| No | 1.025 (0.975-1.077) | 0.341 |  |  | 1.136 (1.058-1.220) | < 0.001 |  |
| Yes | 1.017 (0.962-1.076) | 0.551 |  |  | 1.152 (1.070-1.239) | < 0.001 |  |
| Infant gender |  |  | 0.826 |  |  |  | 0.695 |
| Male | 1.010 (0.960-1.063) | 0.695 |  |  | 1.140 (1.062-1.223) | < 0.001 |  |
| Female | 1.019 (0.965-1.076) | 0.503 |  |  | 1.147 (1.065-1.235) | < 0.001 |  |

^a^ Adjusted for age, ethnicity, occupation type, marital status, blood type, nonprimary status, anaemia status, infant weight, preeclampsia status, vaginitis status, gestational hypertension status, thyroid disease status, temperature, and relative humidity.

## Table S14 Subgroup analysis of the association between NO_3_^-^ exposure in the first to second trimester and GDM risk

| Subgroup | Crude | | |  | Adjusted^a^ | | |
| --- | --- | --- | --- | --- | --- | --- | --- |
|  | OR (95%CI) | *p*-value | *p* for interaction |  | OR (95%CI) | *p*-value | *p* for interaction |
| Age |  |  | 0.312 |  |  |  | 0.269 |
| < 35 years | 0.999 (0.964-1.036) | 0.974 |  |  | 1.275 (1.178-1.381) | < 0.001 |  |
| ≥ 35 years | 0.959 (0.893-1.030) | 0.250 |  |  | 1.153 (0.991-1.341) | 0.066 |  |
| Ethnicity |  |  | 0.956 |  |  |  | 0.970 |
| Han | 0.988 (0.957-1.021) | 0.479 |  |  | 1.240 (1.155-1.331) | < 0.001 |  |
| Other | 0.982 (0.788-1.225) | 0.874 |  |  | 1.130 (0.701-1.823) | 0.616 |  |
| Occupation type |  |  | 0.500 |  |  |  | 0.764 |
| Employee | 0.971 (0.930-1.014) | 0.182 |  |  | 1.216 (1.106-1.336) | < 0.001 |  |
| Freelancer | 1.054 (0.939-1.184) | 0.373 |  |  | 1.339 (1.034-1.734) | 0.027 |  |
| Other | 0.994 (0.944-1.046) | 0.811 |  |  | 1.253 (1.116-1.408) | < 0.001 |  |
| Marital status |  |  | 0.640 |  |  |  | 0.674 |
| Married | 0.990 (0.958-1.022) | 0.528 |  |  | 1.252 (1.166-1.344) | < 0.001 |  |
| Unmarried | 0.943 (0.771-1.152) | 0.565 |  |  | 0.872 (0.541-1.404) | 0.572 |  |
| Blood type |  |  | 0.889 |  |  |  | 0.919 |
| Type A | 0.990 (0.930-1.054) | 0.746 |  |  | 1.318 (1.151-1.509) | < 0.001 |  |
| Type B | 0.965 (0.907-1.027) | 0.266 |  |  | 1.154 (1.002-1.328) | 0.047 |  |
| Type O | 0.984 (0.936-1.035) | 0.529 |  |  | 1.194 (1.070-1.333) | 0.002 |  |
| Type AB | 1.088 (0.964-1.227) | 0.170 |  |  | 1.582 (1.219-2.053) | 0.001 |  |
| Nonprimary status |  |  | 0.314 |  |  |  | 0.411 |
| No | 1.012 (0.969-1.057) | 0.594 |  |  | 1.238 (1.136-1.349) | < 0.001 |  |
| Yes | 0.979 (0.933-1.027) | 0.381 |  |  | 1.263 (1.115-1.430) | < 0.001 |  |
| Anemia status |  |  | 0.320 |  |  |  | 0.225 |
| No | 1.006 (0.964-1.049) | 0.790 |  |  | 1.251 (1.134-1.379) | < 0.001 |  |
| Yes | 0.973 (0.927-1.022) | 0.276 |  |  | 1.231 (1.112-1.362) | < 0.001 |  |
| Infant gender |  |  | 0.729 |  |  |  | 0.684 |
| Male | 0.983 (0.941-1.027) | 0.445 |  |  | 1.241 (1.126-1.367) | < 0.001 |  |
| Female | 0.994 (0.949-1.042) | 0.811 |  |  | 1.240 (1.120-1.373) | < 0.001 |  |

^a^ Adjusted for age, ethnicity, occupation type, marital status, blood type, nonprimary status, anaemia status, infant weight, preeclampsia status, vaginitis status, gestational hypertension status, thyroid disease status, temperature, and relative humidity.

## Table S15 Subgroup analysis of the association between NH_4_^+^ exposure in the first to second trimester and GDM risk

| Subgroup | Crude | | |  | Adjusted^a^ | | |
| --- | --- | --- | --- | --- | --- | --- | --- |
|  | OR (95%CI) | *p*-value | *p* for interaction |  | OR (95%CI) | *p*-value | *p* for interaction |
| Age |  |  | 0.338 |  |  |  | 0.288 |
| < 35 years | 1.005 (0.954-1.059) | 0.848 |  |  | 1.352 (1.220-1.498) | < 0.001 |  |
| ≥ 35 years | 0.950 (0.857-1.053) | 0.329 |  |  | 1.203 (0.991-1.461) | 0.062 |  |
| Ethnicity |  |  | 0.985 |  |  |  | 0.981 |
| Han | 0.989 (0.944-1.036) | 0.637 |  |  | 1.309 (1.194-1.434) | < 0.001 |  |
| Other | 0.992 (0.721-1.364) | 0.960 |  |  | 1.113 (0.600-2.064) | 0.735 |  |
| Occupation type |  |  | 0.436 |  |  |  | 0.663 |
| Employee | 0.961 (0.903-1.024) | 0.219 |  |  | 1.266 (1.122-1.430) | < 0.001 |  |
| Freelancer | 1.085 (0.918-1.282) | 0.338 |  |  | 1.451 (1.037-2.032) | 0.030 |  |
| Other | 0.999 (0.928-1.077) | 0.988 |  |  | 1.337 (1.151-1.553) | < 0.001 |  |
| Marital status |  |  | 0.605 |  |  |  | 0.624 |
| Married | 0.991 (0.946-1.038) | 0.700 |  |  | 1.324 (1.209-1.451) | < 0.001 |  |
| Unmarried | 0.917 (0.686-1.226) | 0.558 |  |  | 0.828 (0.451-1.521) | 0.543 |  |
| Blood type |  |  | 0.796 |  |  |  | 0.840 |
| Type A | 0.997 (0.911-1.091) | 0.948 |  |  | 1.417 (1.191-1.686) | < 0.001 |  |
| Type B | 0.952 (0.871-1.041) | 0.282 |  |  | 1.184 (0.987-1.421) | 0.068 |  |
| Type O | 0.982 (0.913-1.055) | 0.620 |  |  | 1.251 (1.086-1.442) | 0.002 |  |
| Type AB | 1.134 (0.954-1.349) | 0.154 |  |  | 1.770 (1.263-2.480) | 0.001 |  |
| Nonprimary status |  |  | 0.358 |  |  |  | 0.416 |
| No | 1.023 (0.961-1.089) | 0.475 |  |  | 1.306 (1.168-1.459) | < 0.001 |  |
| Yes | 0.979 (0.913-1.050) | 0.551 |  |  | 1.336 (1.139-1.566) | < 0.001 |  |
| Anemia status |  |  | 0.435 |  |  |  | 0.299 |
| No | 1.011 (0.951-1.075) | 0.735 |  |  | 1.307 (1.153-1.483) | < 0.001 |  |
| Yes | 0.974 (0.908-1.044) | 0.459 |  |  | 1.31 (1.15-1.491) | < 0.001 |  |
| Infant gender |  |  | 0.772 |  |  |  | 0.711 |
| Male | 0.983 (0.923-1.047) | 0.587 |  |  | 1.311 (1.157-1.485) | < 0.001 |  |
| Female | 0.996 (0.931-1.066) | 0.911 |  |  | 1.305 (1.145-1.487) | < 0.001 |  |

^a^ Adjusted for age, ethnicity, occupation type, marital status, blood type, nonprimary status, anaemia status, infant weight, preeclampsia status, vaginitis status, gestational hypertension status, thyroid disease status, temperature, and relative humidity.

## Table S16 Subgroup analysis of the association between OM exposure in the first to second trimester and GDM risk

| Subgroup | Crude | | |  | Adjusted^a^ | | |
| --- | --- | --- | --- | --- | --- | --- | --- |
|  | OR (95%CI) | *p*-value | *p* for interaction |  | OR (95%CI) | *p*-value | *p* for interaction |
| Age |  |  | 0.361 |  |  |  | 0.281 |
| < 35 years | 1.018 (0.991-1.046) | 0.193 |  |  | 1.126 (1.081-1.172) | < 0.001 |  |
| ≥ 35 years | 0.990 (0.938-1.045) | 0.713 |  |  | 1.072 (0.994-1.155) | 0.071 |  |
| Occupation type |  |  | 0.740 |  |  |  | 0.761 |
| Employee | 1.008 (0.983-1.033) | 0.542 |  |  | 1.112 (1.073-1.152) | < 0.001 |  |
| Freelancer | 1.036 (0.880-1.220) | 0.669 |  |  | 1.006 (0.787-1.285) | 0.964 |  |
| Other |  |  | 0.285 |  |  |  | 0.501 |
| Marital status | 0.990 (0.958-1.024) | 0.559 |  |  | 1.096 (1.045-1.149) | < 0.001 |  |
| Married | 1.047 (0.961-1.141) | 0.295 |  |  | 1.138 (0.997-1.300) | 0.056 |  |
| Unmarried | 1.018 (0.979-1.059) | 0.365 |  |  | 1.126 (1.062-1.195) | < 0.001 |  |
| Blood type |  |  | 0.414 |  |  |  | 0.404 |
| Type A | 1.010 (0.985-1.035) | 0.447 |  |  | 1.117 (1.078-1.158) | < 0.001 |  |
| Type B | 0.945 (0.809-1.105) | 0.479 |  |  | 0.883 (0.696-1.120) | 0.304 |  |
| Type O |  |  | 0.500 |  |  |  | 0.569 |
| Type AB | 1.024 (0.977-1.073) | 0.327 |  |  | 1.147 (1.071-1.227) | < 0.001 |  |
| Nonprimary status | 0.981 (0.936-1.029) | 0.437 |  |  | 1.061 (0.988-1.139) | 0.105 |  |
| No | 1.003 (0.965-1.042) | 0.888 |  |  | 1.097 (1.037-1.159) | 0.001 |  |
| Yes | 1.078 (0.984-1.180) | 0.105 |  |  | 1.243 (1.088-1.421) | 0.001 |  |
| Anemia status |  |  | 0.522 |  |  |  | 0.473 |
| No | 1.027 (0.994-1.060) | 0.107 |  |  | 1.112 (1.064-1.162) | < 0.001 |  |
| Yes | 1.010 (0.973-1.049) | 0.598 |  |  | 1.112 (1.046-1.182) | 0.001 |  |
| Infant gender |  |  | 0.614 |  |  |  | 0.406 |
| Male | 1.018 (0.986-1.052) | 0.269 |  |  | 1.113 (1.059-1.169) | < 0.001 |  |
| Female | 1.006 (0.970-1.043) | 0.759 |  |  | 1.108 (1.053-1.167) | < 0.001 |  |
| Infant gender |  |  | 0.808 |  |  |  | 0.668 |
| Male | 1.005 (0.973-1.039) | 0.748 |  |  | 1.107 (1.054-1.162) | < 0.001 |  |
| Female | 1.012 (0.976-1.048) | 0.526 |  |  | 1.114 (1.058-1.173) | < 0.001 |  |

^a^ Adjusted for age, ethnicity, occupation type, marital status, blood type, nonprimary status, anaemia status, infant weight, preeclampsia status, vaginitis status, gestational hypertension status, thyroid disease status, temperature, and relative humidity.

## Table S17 Subgroup analysis of the association between BC exposure in the first to second trimester and GDM risk

| Subgroup | Crude | | |  | Adjusted^a^ | | |
| --- | --- | --- | --- | --- | --- | --- | --- |
|  | OR (95%CI) | *p*-value | *p* for interaction |  | OR (95%CI) | *p*-value | *p* for interaction |
| Age |  |  | 0.368 |  |  |  | 0.280 |
| < 35 years | 1.134 (0.977-1.315) | 0.098 |  |  | 1.770 (1.443-2.171) | < 0.001 |  |
| ≥ 35 years | 0.975 (0.727-1.307) | 0.863 |  |  | 1.393 (0.955-2.031) | 0.086 |  |
| Ethnicity |  |  | 0.675 |  |  |  | 0.706 |
| Han | 1.069 (0.936-1.221) | 0.322 |  |  | 1.664 (1.389-1.994) | < 0.001 |  |
| Other | 1.295 (0.534-3.142) | 0.568 |  |  | 1.002 (0.290-3.463) | 0.997 |  |
| Occupation type |  |  | 0.260 |  |  |  | 0.459 |
| Employee | 0.973 (0.813-1.164) | 0.762 |  |  | 1.551 (1.222-1.970) | < 0.001 |  |
| Freelancer | 1.271 (0.796-2.029) | 0.315 |  |  | 1.809 (0.921-3.555) | 0.085 |  |
| Other | 1.142 (0.922-1.415) | 0.224 |  |  | 1.791 (1.329-2.414) | < 0.001 |  |
| Marital status |  |  | 0.350 |  |  |  | 0.330 |
| Married | 1.082 (0.947-1.236) | 0.245 |  |  | 1.706 (1.423-2.044) | < 0.001 |  |
| Unmarried | 0.719 (0.308-1.676) | 0.445 |  |  | 0.528 (0.163-1.713) | 0.288 |  |
| Blood type |  |  | 0.442 |  |  |  | 0.515 |
| Type A | 1.186 (0.918-1.531) | 0.192 |  |  | 1.955 (1.387-2.756) | < 0.001 |  |
| Type B | 0.915 (0.708-1.183) | 0.498 |  |  | 1.292 (0.902-1.851) | 0.162 |  |
| Type O | 1.042 (0.848-1.282) | 0.694 |  |  | 1.569 (1.187-2.075) | 0.002 |  |
| Type AB | 1.531 (0.935-2.506) | 0.090 |  |  | 2.871 (1.456-5.662) | 0.002 |  |
| Nonprimary status |  |  | 0.648 |  |  |  | 0.461 |
| No | 1.181 (0.991-1.406) | 0.062 |  |  | 1.676 (1.340-2.096) | < 0.001 |  |
| Yes | 1.108 (0.900-1.364) | 0.332 |  |  | 1.644 (1.214-2.227) | 0.001 |  |
| Anemia status |  |  | 0.735 |  |  |  | 0.505 |
| No | 1.124 (0.942-1.342) | 0.193 |  |  | 1.654 (1.292-2.118) | < 0.001 |  |
| Yes | 1.074 (0.882-1.309) | 0.477 |  |  | 1.657 (1.280-2.145) | < 0.001 |  |
| Infant gender |  |  | 0.804 |  |  |  | 0.643 |
| Male | 1.057 (0.883-1.266) | 0.543 |  |  | 1.617 (1.263-2.070) | < 0.001 |  |
| Female | 1.093 (0.902-1.324) | 0.363 |  |  | 1.693 (1.307-2.192) | < 0.001 |  |

^a^ Adjusted for age, ethnicity, occupation type, marital status, blood type, nonprimary status, anaemia status, infant weight, preeclampsia status, vaginitis status, gestational hypertension status, thyroid disease status, temperature, and relative humidity.
